# Network-Based Data Integration for Selecting Candidate Virulence Associated Proteins in the Cereal Infecting Fungus *Fusarium graminearum*

**DOI:** 10.1371/journal.pone.0067926

**Published:** 2013-07-04

**Authors:** Artem Lysenko, Martin Urban, Laura Bennett, Sophia Tsoka, Elzbieta Janowska-Sejda, Chris J. Rawlings, Kim E. Hammond-Kosack, Mansoor Saqi

**Affiliations:** 1 Department of Computational and Systems Biology, Rothamsted Research, Harpenden, United Kingdom; 2 Department of Informatics, School of Natural and Mathematical Sciences, Kings College London, Strand, London, United Kingdom; 3 Department of Plant Biology and Crop Science, Rothamsted Research, Harpenden, United Kingdom; Seoul National University, Republic of Korea

## Abstract

The identification of virulence genes in plant pathogenic fungi is important for understanding the infection process, host range and for developing control strategies. The analysis of already verified virulence genes in phytopathogenic fungi in the context of integrated functional networks can give clues about the underlying mechanisms and pathways directly or indirectly linked to fungal pathogenicity and can suggest new candidates for further experimental investigation, using a ‘guilt by association’ approach. Here we study 133 genes in the globally important Ascomycete fungus *Fusarium graminearum* that have been experimentally tested for their involvement in virulence. An integrated network that combines information from gene co-expression, predicted protein-protein interactions and sequence similarity was employed and, using 100 genes known to be required for virulence, we found a total of 215 new proteins potentially associated with virulence of which 29 are annotated as hypothetical proteins. The majority of these potential virulence genes are located in chromosomal regions known to have a low recombination frequency. We have also explored the taxonomic diversity of these candidates and found 25 sequences, which are likely to be fungal specific. We discuss the biological relevance of a few of the potentially novel virulence associated genes in detail. The analysis of already verified virulence genes in phytopathogenic fungi in the context of integrated functional networks can give clues about the underlying mechanisms and pathways directly or indirectly linked to fungal pathogenicity and can suggest new candidates for further experimental investigation, using a ‘guilt by association’ approach.

## Introduction

The Ascomycete fungus *Fusarium graminearum* (teleomorph *Gibberella zeae*) is a major pathogen of wheat causing *Fusarium* ear blight, Fusarium head blight or *Fusarium* head scab disease [1], [2] (www.scabusa.org). As wheat accounts for 32% of global cereal production and provides 20% of the world’s calorific intake (www.fao.org), control strategies for *Fusarium* infection are important for food security. *Fusarium graminearum* can also infect the floral tissue of numerous other cereal species, including maize, barley, triticale, rice and oats [1]. Although affecting yield, *Fusarium* infection often leads to reduced grain quality and to contamination of the grain with various mycotoxins, in particular the ß-type trichothecene deoxynivalenol (DON) and its acetylated derivatives (15A-DON and 3A-DON), which may make the grain unsafe for human and/or animal consumption [3].

The genome sequence of *Fusarium graminearum* [4] is predicted to code for 13,332 proteins and further revisions to the identification of open reading frames and annotation are in progress [5], [6]. As a result of the analysis of a genetic cross between the sequenced strain and another strain, the *F. graminearum* genome is recognised to contain regions of high recombination in both sub-telomeric and central chromosome regions interspersed with longer regions with low or no genetic recombination. Genes shared between different Fusarium species are primarily located in the low and no recombination regions [4]. Particular genes in *F. graminearum,* other Fusarium species and other plant fungal pathogens have been investigated experimentally for their contribution to pathogenicity or virulence, i.e. their qualitative or quantitative effect of the disease causing ability of a microbe. Typically these experiments involve stable gene disruption/gene deletion in the pathogen and observation of the resulting infection phenotype in one or more host plant systems. Already a large number of *F. graminearum* genes have been tested and published, of which 100 were found to alter virulence and 33 had no effect on the interaction tested at the time of writing this article [7] and **Table S1**)**.** Several of these *F. graminearum* virulence genes are unique to this species or restricted to closely related Fusarium species whilst others genes are also required for virulence in other plant and/or animal infecting microbes. To assist comparative studies, the functions in numerous other pathosystems of pathogenicity and virulence associated genes has been catalogued in the Pathogen-Host Interactions database called PHI-base [8]–[10], accessible at www.phi-base.org. This is an expertly curated database for ∼1000 pathogen-host interactions. The plant, animal, fungal, oomycete and/or bacteria entries in PHI-base are extracted from the scientific literature by domain experts and therefore describe experimentally tested interactions, for example the effect of a given gene disruption experiment in a given pathogen, on a particular host. Importantly PHI-base also details those tested genes, which had no effect on pathogenicity.

In order to understand how particular genes and their gene products may contribute to the pathogenic process it is necessary to explore the biological context of these genes. Approaches that involve placing these genes within various relationship networks provide a useful starting point. The relationships can include, for example, gene co-expression, known or predicted protein-protein interactions, and sequence similarity (see for example [11] ). Previously, a predicted protein-protein interaction (PPI) network has been used to predict pathogenicity genes in *Fusarium graminearum* [12]. This ‘guilt by association’ approach [13] was used to examine those proteins in a predicted PPI network [14] that have at least two known pathogen associated genes as nearest neighbours with additional filtering of candidates using some of the available *in planta* and *in vitro* gene expression data available from a comprehensive data source called PLEXdb [15]. The Liu et al. network analysis used an initial list of 49 *F. graminearum* gene sequences available in PHI-base. A total of 39 potential virulence associated proteins were identified, of which nine have now been connected to virulence through experimentation (reviewed in [7]).

Here we extend the study of [12] by using an integrated network that includes co-expression information and sequence similarity in addition to the core predicted PPI network [14] as well as a larger set of known *Fusarium graminearum* virulence associated genes. The aim of this study was two-fold: firstly to predict additional *F. graminearum* virulence associated genes that could then become targets for experimental analysis and secondly to enable the biological context of the predictions to be explored. As our starting point, we have used the set of verified virulence (VV) genes taken from the pathogen host interaction database PHI-base (version 3.3) as well as manual curation of the recent literature on *Fusarium graminearum* pathogenicity in order to include entries not yet in PHI-base 3.3. The data integration has been carried out using the Ondex data integration and visualisation system [16], [17] which allows the integrated network to be explored manually. The filtering tools in the Ondex system allow the effects of inclusion or exclusion of various evidence types on the predictions to be inspected. We discuss in detail the biological plausibility of some of the predictions. The predictions in the context of the entire network have been made available for use by the community. We acknowledge that the term virulence associated genes/proteins can be interpreted in a number of ways – the candidates we have identified may be involved in some part of the virulence process but not necessarily be directly involved (for example, an effector protein) and could be seen as system components [18].

## Results

### Predictions Made with the Integrated Network

We constructed an integrated network for *Fusarium graminearum* using information from protein sequence similarity, gene co-expression and predicted protein interactions (PPI). The coexpression links were created between nodes representing proteins if the genes encoding them were found to be coexpressed. We have previously described the disjoint and overlapping community structure of the integrated network in [19]. Here we use the network for prediction of potential new virulence associated proteins.

**Table 1** shows the graph topological properties, calculated with the NetworkX package [20], of the three constituent networks, as well as an integrated network, which uses information from all three constituent networks. The sequence similarity network has a large number of connected components (subgraphs in which any two nodes are connected by a path of edges) and a high transitivity measure (suggesting more tightly connected structures, i.e a more ‘clique-like’ structure). These properties most likely reflect the grouping of the proteins into sequence similar groups. The predicted protein interaction network from [14] also has a high transitivity suggesting a more ‘clique like’ structure, which may be an indication of predicted protein complexes, although this structure may be affected by the way in which data from some experiments is interpreted and represented as binary interactions in different PPI data sources [17].

**Table 1:** Comparison of the global properties of the four predicted networks.

Network type	Nodes	Edges	VV seeds leading to predictions	Connected components (CC)	Size of largest CC	Transitivity	Predictions
**Sequence network**	6349	27807	19 (12)	1155	625	0.69	100 (61)
**Core PPI ** [**14**]	3459	24348	30 (21)	111	2995	0.85	79 (54)
**Co-expression**	3654	33272	18 (13)	159	3239	0.42	47 (14)
**Integrated**	9521	80997	60 (50)	439	8364	0.52	215 (120)

Global properties of the three constituent networks and the integrated network. The sequence similarity network excludes nodes with no edges (‘orphan’ proteins with no sequence similarity matches). Column 4 is the number of verified virulence (VV) seeds involved in the predictions, using the rule that a node must be connected to at least 2 seeds to be a prediction (in brackets are the corresponding numbers if we require connection to at least 3 seeds); Column 8 gives the number of predictions (in brackets are the corresponding prediction counts if we require a more stringent rule i. e. a node must be connected to at least 3 seeds to be a prediction).

The prediction of virulence associated proteins was carried out in the Ondex software by the implementation of a new plug-in, as described in the Methods section. Following [12] a node in the network was labelled as a predicted virulence associated protein if it was a nearest neighbour to at least two VV seeds. Fewer VV seeds were involved in predictions in the co-expression network than the PPI network (18 seeds as compared to 30). As expected, the integrated network was the largest and had the greatest number of VV seeds which were involved in predictions (60).

This approach resulted in 215 predictions in the integrated network, which was considerably more than could be predicted from any of the individual constituent networks: using only the sequence similarity based network leads to 100 predictions, the coexpression network yields 47 predictions and the predicted PPI network of Zhao et al (2009) 79 predictions. The 215 predictions (**Table S2**) contain 29 proteins annotated as hypothetical protein in the *Fusarium graminearum* database [6]. The predictions made on the basis of PPI links to the VV seeds may reflect an ancient species conserved sub network, because the *Fusarium* PPI network originally described by [14] had been developed using information from six eukaryotic species and one prokaryotic species, which are all non-pathogenic, namely, budding and fission yeasts, human, mouse, fly, worm and *E. coli*. The predictions made on the basis of co-expression links could potentially represent, either a fungal taxon restricted, but conserved network, a *Fusarium graminearum* specific network or again be part of an ancient species conserved network. The complete list of all predictions and the seeds they are connected to is available as **Table S3**. We have also included what proportion of all edges for each of the predicted nodes are linked to seeds. Although, it would be reasonable to assume that a higher proportion would indicate a more certain prediction, the small numbers of available seeds did not allow us to explore this further as part of this study.

Some predictions were made on the basis of the node being a nearest neighbour to a larger number of VV seeds and these may represent more confident predictions. **Table 2** shows the distribution of the number of seeds to which each predicted virulence associated node is connected in the integrated network as well as in the three constituent networks.

**Table 2:** Predicting virulence nodes based on the seed numbers connected within the local neighbourhood.

	Number of nodes connected to a given number of seeds			
Number of seeds	Integrated	Protein-protein interaction	Co-expression	Sequence similarity
**2**	95	25	33	39
**3**	58	48	11	23
**4**	32	6	3	25
**5**	23	0	0	12
**6**	3	0	0	1
**7**	3	0	0	0
**8**	1	0	0	0

The number of seeds to which each predicted virulence node is connected, in the four networks is shown. A node linked to 2 or more seed nodes is termed a prediction. Some predictions have links to multiple seeds.

The method for selection of candidate virulence associated proteins based on the network neighbourhood of the proteins previously reported to be important for infection and disease formation was reported by the study of [12]. However, the original study did not validate the underlying assumption that proteins important for virulence are in fact more likely to be connected to other proteins with similar properties. To test this assumption, the node labels were permuted 10000 times to give an estimate of how likely any protein annotated to be involved in virulence is to be connected with at least two others. As shown in **Table 3**, we have observed that the probability is significantly higher than would be expected by chance for sequence similarity and protein-protein interaction networks, but not so for the co-expression network. This result can be taken as an indication that the selection strategy used in this work can be used to reveal the most relevant candidate proteins.

**Table 3:** The probability that a verified virulence (VV)seed is connected to at least 2 others by chance.

Network type	Seeds connected to 2 or more other seeds	p-value
**Integrated**	13	0.0001
**Protein-protein interaction**	4	0.0186
**Co-expression**	3	0.1172
**Sequence similarity**	7	7.00E-04

We have described the community structure of the largest connected component of the integrated network in another study [19]. First a series of disjoint (non-overlapping) communities of the network were detected using the Louvain method [21] (which optimises a measure known as modularity [22]. Modularity optimisation is a widely accepted method for community structure detection and has proven its utility in many biological applications and in particular has found functionally coherent communities in PPI networks [23], [24]. These disjoint communities were then transformed into overlapping communities through the application of a mathematical programming method, which allows nodes making connections across community borders to be multi-clustered according to the optimisation of another metric known as community strength [19]. In the transformation from disjoint to overlapping communities, the extent of overlapping, i.e. the number of proteins that belong to multiple communities, is controlled by a parameter r. In general, the multi-clustered proteins were found to have a higher connectivity and higher multi-functionality based on Gene Ontology (GO) annotations than proteins belonging to only one module. We found that overall the verified virulence proteins did not appear to show a tendency to belong to multiple communities although one case was noted (r = 0.4), where nearly half (49.3%) of the VV proteins belonged to more than one community. We are aware that the small number of VV proteins makes it difficult to ascribe biological significance to these results. We explore here whether the 215 predictions also exhibit the same behaviour. We find that 164 out of the 215 are in the largest module (of size 1951 nodes), which also contains 33 seeds. This module was previously shown to be significantly enriched for VV proteins, and therefore, it makes sense that a large number of the predictions also belong to this community due the nature of guilt-by-association. We now consider the module membership of the predictions to determine whether they tend to belong to more than one module. We find that according to the Fisher’s exact test, a significant proportion of the predicted proteins do belong to more than one module (in the range 0.4≤ r ≤0.9). This may be due to the fact that multi-clustered proteins tend to be more connected than proteins belonging to only one module and therefore have a higher chance of being connected to the VV proteins. However, it may also indicate that the proteins predicted to be virulence associated may have a tendancy to be multi-functional.

We also compared the length distributions of the set of 215 predicted virulence associated proteins with the length distribution of all the proteins in the *F graminearum* genome and find that average lengths of proteins in the predictions and the seeds subset are significantly greater than all the other *F. graminearum* predicted proteins (n = 12,984) (Student’s t-test, t = 4.49, d.f. = 79.30, p<0.01 for seeds vs. other and t = 4.03, d.f. = 225.48, p<0.01 for predictions vs. other). The larger mean size of the VV seeds compared to the ‘others’ category has arisen purely as a result of the initial protein types selected by the global fusarium community for functional experimentation. The underlying reasons for the increased length of the predicted virulence associated proteins compared to all the other proteins predicted from the *F. graminearum* sequenced genome is currently unclear. However, this analysis clearly indicates that small protein sequences are under-represented in the predictions. The 29 hypothetical proteins predicted have the size range 69 to 1399 amino acids (aa) (mean 527 aa), with only 4 proteins having length under 200 aa (FGSG_01228 (186), FGSG_00536 (116), FGSG_01888 (69), FGSG_08359 (178). We also explored the overall predictive power of the four different networks (**Table S4**). This analysis revealed a marked improvement over the random model. However, the small number of positive and negative examples are insufficient to make an accurate estimate for either the sensitivity or specificity values.

### Taxonomic Diversity of the Predictions

**Figure 1:**
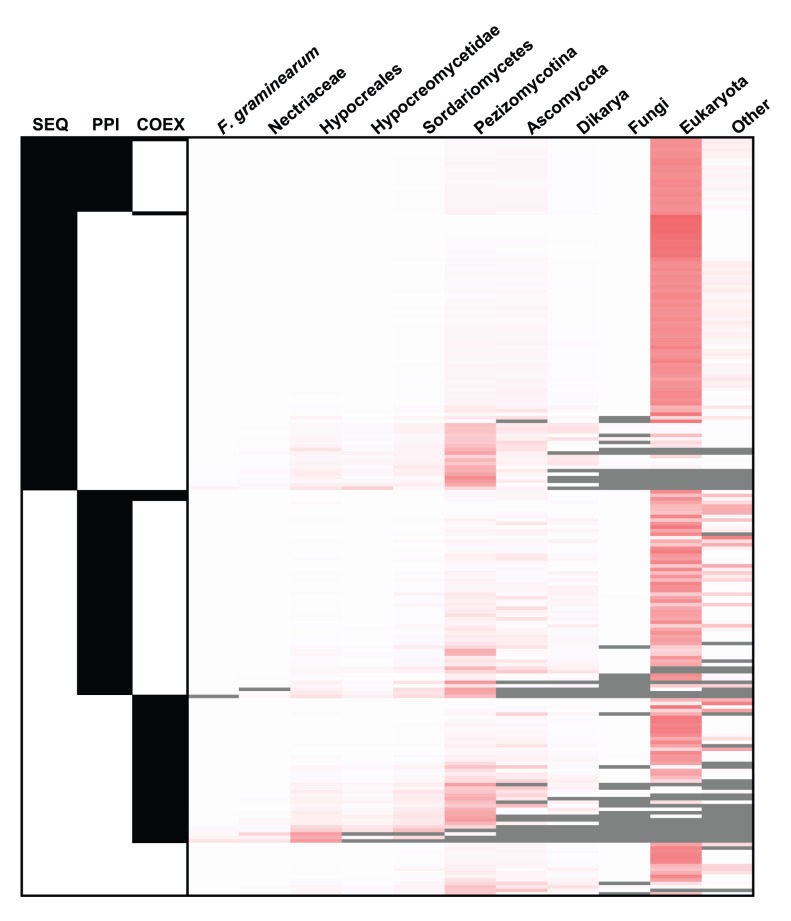
Heat map displaying the taxonomic distribution for each of the predicted virulence associated proteins. Each row provides the information for one sequence. The left hand three columns (SEQ, PPI, COEX) indicate the network in which the prediction could be made (black). For the bottom 15 rows only the integrated network provides the prediction. The right hand heatmap shows the proportional distribution of all BLAST hits from the 215 predictions to the NCBI nr database (white – lowest, red - highest) across the taxonomical levels. All hits were counted once, at the lowest possible level of taxonomical specificity. The grey colour shows cases where there were no hits at a particular taxonomic level. See **Table S2** for the detailed results for each individual FGSG protein.

The taxonomic diversity of the 215 predicted virulence associated proteins was explored by matching the sequences against the non-redundant database at NCBI (www.ncbi.nlm.nih.gov) so as to obtain an indication of which of the predictions is *Fusarium* or fungal specific. This distribution is represented as a heatmap (**Figure 1**), and the details for each FGSG gene are shown in **Table S5.**

Twenty-five of the predictions are specific up to the level of fungi, whilst 15 are specific up to the level of *Ascomycota*. The FGSG_10808 (a conserved hypothetical protein) and FGSG_03534 (trichothecene 15-O-acetyltransferase) are highly specific to the level of *Hypocreales*. This analysis also highlights that there are 15 predictions unique to the integrated network. Of these six are found to have a taxonomic distribution beyond eukaryotes. Overall, this analysis confirms that the predictions present within each network are for sequences shared with many other eukaryotic species as well as in some case prokaryote species.

### Exploring Predictions from Connections to Multiple Seeds

The requirement for a node in the network to be a candidate for virulence was connected to at least two seed VV nodes. As can be seen from **Table 2**, some nodes were connected to a greater number of seeds and these may be suggestive of stronger predictions. One prediction (FGSG_06878) was made on the basis of 8 links to seed proteins. The annotations of the seeds that contributed to the prediction of this protein are given in **Table 4** **.**

**Table 4:** The prediction of FGSG_06878 as a virulence factor with links to 8 seeds.

FGSG_06878 (probable CMK1 - Ca2+/calmodulin-dependent ser/thr protein kinase type I) is linked by	Seeds on which the prediction is based with phenotype [], and MIPS annotation.Phenotype symbols are rv = reduced virulence, lp = loss of pathogenicity		
Predicted PPI to:	**FGSG_09903** *(ste7)* [lp], Probable map kinase kinase	**FGSG_10313** [rv] *(mgv1)* (MGV1 map kinase)	**FGSG_06385** *(map1)* [lp] (FMK1 pathogenicity map kinase 1
Co-expression to:	**FGSG_08737** *(GzOB031)* [rv] Probable woronin body major protein precursor	**FGSG_01964** *(CHS5)* [rv] Probable chitin synthase	**FGSG_00385** *(GzHMG002)* [rv] probable NHP6B - nonhistone chromosomal protein
Sequence similarity to:	**FGSG_09897** *(snf1)* [rv] probable serine/threonine protein kinase	**FGSG_06385** *(map1)* [lp] (FMK1 pathogenicity map kinase 1)	**FGSG_16491** *(fst11)* [lp]] related to NRC-1 MAPKK kinase

This prediction FGSG_06878 was confirmed to be a virulence protein in the recent paper of [21-Wang et al.]. Note that prediction FGSG_06878 is linked to seed FGSG_06385 by both predicted PPI and sequence similarity information. In planta phenotypes are rv, reduced virulence, a quantitative reduction in disease causing ability and the more stringent lp, indicating loss of pathogenicity where disease establishment is aborted.

**Figure 2:**
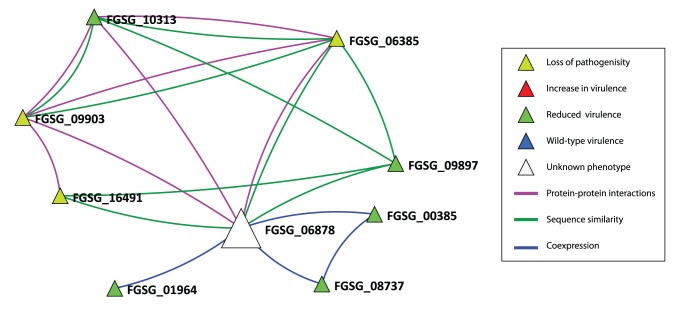
The local neighbourhood for the predicted virulence gene FGSG_06878. The neighbourhood of FGSG_06878 (prediction -large white triangle) and these 8 seed proteins to which it is linked, visualised with Ondex [16]. The magenta coloured edges predicted PPI information, blue edges predicted co-expression information and the green coloured edges predict sequence similarity information.

To facilitate the detailed analysis of the network neighbourhood of the predicted virulence associated nodes of interest, the Ondex visualisation tool was used (**Figure 2** **, Figure S1** for complete neighourhood). These Ondex displays permit the experimenter to explore simultaneously the details associated with each node as well as the origin of the different types of source information via inspection of the colour of each edge connecting the seed to the predicted node.

The prediction FGSG_06878 is linked to 5 seeds with associated phenotype ‘reduced virulence’, namely FGSG_10313 *(MGV1)*, FGSG_00385, FGSG_08737, FGSG_01964, FGSG_09897 *(SNF1)*, and 3 seeds (FGSG_09903 *(PKAR)* and FGSG_06385 *(FMK1)* and FGSG_16491 *(FST11)* with associated phenotype ‘loss of pathogenicity’. This predicted virulence associated protein, FGSG_06878 is annotated in GenRE database [6] as a “probable CMK1 - Ca2+/calmodulin-dependent ser/thr protein kinase type I”. The prediction and the seeds from which this prediction was inferred are shown in **Figure 2**. Evidence for crosstalk between Map kinase (MAPK) and calcium-calmodulin dependent signalling leading to the activation of transcripton factors was established earlier and was recently reviewed for several plant human pathogenic fungi. [25]. A recent gene deletion study by [26] confirmed a reduced virulence phenotype for FGSG_06878.

The full details of three other predictions that have links to 7 seeds are given in **Table S6** and the immediate networks are displayed in **Figures S2, S3 and S4.** In each case, at least one of the seeds is annotated to be a transcription factor and the prediction is made from information obtained from only two of the constituent networks.

### Other Examples of Specific Predictions

In total, this integrated network analysis has predicted 215 potential virulence associated proteins. For illustrative purposes three very different types of predictive example are discussed in detail. The first example was selected because it illustrates the effect of multiple complementary information types contributing to the prediction, the second because a protein unique to *F. graminearum* was predicted and the third example reveals that a network study can identify a specific class of proteins required for virulence, but is unable to pin-point the specific member of a multigene family.

### Example 1: Prediction of FGSG_00559 with a Role in Intracellular Signalling Modulation

**Figure 3:**
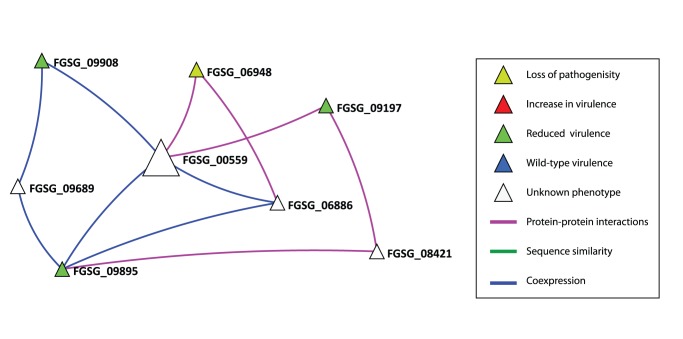
The local neighbourhood for the predicted virulence gene FGSG_00559. The immediate neighbourhood in the integrated network within which the predicted virulence associated protein FGSG_00559 resides (large white triangle). Shown are the types of links between the predictions and the seeds. Magenta coloured edges predicted PPI information and blue edges predicted co-expression information. The various node colours of the seeds as shown in the legend indicate the experimentally determined outcomes. There are 3 additional virulence predictions in this neighbourhood (small white triangles).

Within the integrated network, the protein coded for by the gene FGSG_00559 is predicted on the basis of links to four VV proteins. Two of these links come from the predicted PPI information (magenta edges in **Figure 3**), namely links to FGSG_06948 (*Gzscp*, loss of pathogenicity, related to tetratricopeptide repeat protein tpr1) and FGSG_09197 (*HMR1*, reduced virulence, probable 3-hydroxy-3-methylglutaryl-coenzyme A reductase), whilst two links to other proteins are from co-expression information (blue edges), namely links to FGSG_09895 (*NTH1*, reduced virulence, probable a neutral trehalase (alpha,alpha-trehalose glucohydrolase)) and FGSG_09908 (*PKAR*, reduced virulence, probable cAMP-dependent protein kinase regulatory chain. FGSG_00559 is annotated in the MIPS GenRE database [6] as a probable 26S proteasome regulatory subunit YTA3. Two of these seed proteins FGSG_09895 and FGSG_09908 reside within close physical proximity in the genome, in a micro-region of virulence genes recently identified using a genome landscape scanning – reverse genetics approach [27], [28]. Other predictions included in this network neighbourhood, involving at least two of the same seed proteins include FGSG_06886 a probable 20S core proteasome subunit PRE2, FGSG_09689 a probable ubiquitin-protein ligase (E1-like ubiquitin-activating enzyme) and FGSG_08421 a conserved hypothetical protein. This neighbourhood is highly likely to be involved in co-ordinating two different types of intracellular signalling and possibly involves the degradation of specific signalling components within the proteasome. All the genes in this network neighbourhood were found to reside in regions of either very low or no genetic recombination within the genome [27] and these sequences are found in many fungal and other eukaryotic species (**Table S5**).

### Example 2: Prediction of FGSG_00071 Includes Links to Seeds with Opposite Effects

**Figure 4:**
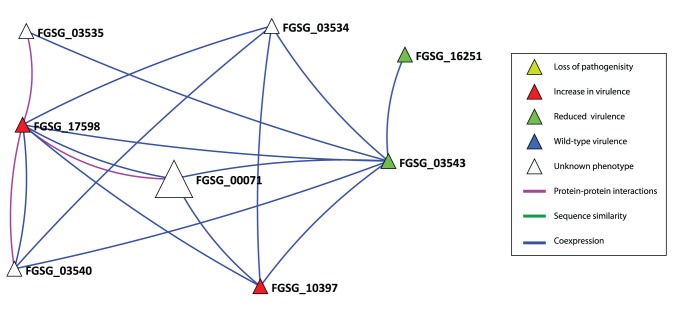
The local neighbourhood for the predicted virulence gene FGSG_00071 *(TRI1)*. Gene IDs are: FGSG_03543 *(TRI14)*, FGSG_10397 *(CLM1)*, FGSG_17598 (related to O-methyl sterigmatocystin oxidoreductase), FGSG_03535 *(TRI4)*, FGSG_03534 *(TRI3)*, FGSG_16251 *(TRI6)*, FGSG_03540 *(TRI11).*

The protein coded for by the gene FGSG_00071 *(TRI1)* is predicted on the basis of links to three VV proteins (**Figure 4**), namely FGSG_16251 (reduced virulence, TRI6, transcription factor) [29], FGSG_03543 (reduced virulence, TRI14, putative trichothecene biosynthesis protein [30] and FGSG_10397 (increase in virulence, CLM1, longiborneol synthetase [31] and FGSG_17598 (recently renamed by MIPS). Previously this gene sequence had been functionally tested as gene FGSG_00007 (increased virulence, cytochrome P450 monooxygenase, DON biosynthesis) [32]. The three other *TRI* genes in this network neighbourhood, namely *TRI3* (FGSG_03534), *TRI4* (FGSG_03535), TRI11 (FGSG_03540) genes are all located within the main trichothecene (*TRI*) biosynthetic cluster, which is in the middle of chromosome 2 in a region of moderately high genetic recombination. These three *TRI* genes are either suggested or have been shown experimentally in *F. graminearum* to code for key steps in the synthesis of various trichothecene mycotoxins, required for deoxyvalenol (DON) and its acetylated derivatives [33].

TRI1 and FGSG_00007/FGSG_17598 are located towards the left end of Chromosome 1, in the region of very high recombination. FGSG_00007/FGSG_17598 is highly expressed under DON inducing conditions. FGSG_17598 is annotated by GenRE as ‘related to O-methylsterigmatocystin oxidoreductase’, but its detailed function is currently unknown.

FGSG_10397 is located in a region of very low recombination at the other end of chromosome 1 and is required for the biosynthesis of a different secondary metabolite, namely Culmorin, when grown under in vitro conditions [31]. In a second study, ([24]-Gardiner et al.) revealed that deletion of FGSG_10397 led to elevated DON mycotoxin production and hence enhanced virulence. However, the level of Culmorin was not reported in the second study.

The predicted virulence node FGSG_00071, is annotated by MIPS as ‘TRI1 cytochrome P450 monooxygenase’. A gene disruption mutant in *F. graminearum* was shown to accumulate calonectrin compounds, and no longer produced 15-acetyldeoxynivalenol [34], however the *in planta* phenotype of this mutant strain has not been reported.

Various *TRI* genes are highly expressed during the symptomless phase of wheat ear colonisation when the fungal hyphae are exclusively extracellularly colonising and are in low abundance [35]. This network neighbourhood which contains conflicting experimental results (both enhanced and reduced virulence phenotypes) appears to be involved in both positively and negatively regulating the production of the trichothecene mycotoxin deoyxnivalenol and its acetylated derivatives as well as one other unrelated secondary metabolite, Culmorin, in response to different external stimuli. Most of the *TRI* genes in *F. graminearum* are highly taxon specific**.** This virulence prediction was made on the basis of two co-expression links and one protein interaction link and suggests value in combining multiple data sources (**Figure 4**). The predicted virulence node FGSG_00071 is specific up to the level of *F. graminearum* (**Table S5).**

### Example 3: Prediction of Two Non Pathogenicity Associated Seeds as Potential Candidates for Virulence

**Figure 5:**
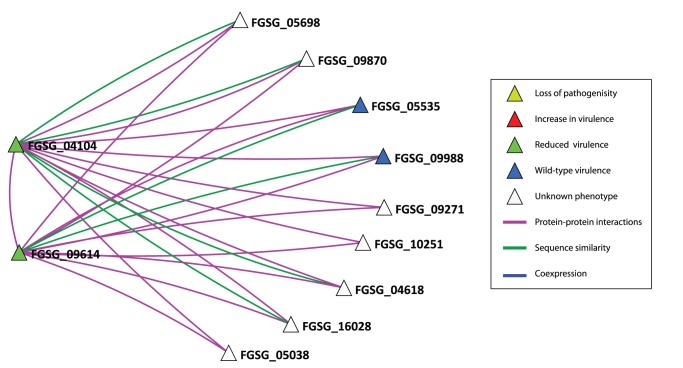
The neighbourhood of FGSG_05535 and FGSG_09988. Although connected to the two seed proteins FGSG_09614 *(GPA2)* and FGSG_04104 *(GPB1),* experimental evidence in barley suggests that the two predictions 05535 and 09988 are dispensable for pathogenicity [36]. Genetic redundancy is suggested to explain this fact. (FGSG_05698: probable *CPC2* protein, FGSG_09870: probable *CPC2* protein; FGSG_09271: probable *SEC13* - protein transport protein; FGSG_10251: probable *LST8* protein; FGSG_04618: related to vegetatible incompatibility protein *HET-E-1*; FGSG_16028: probable U5 snRNP-specific 40 kD protein (novel WD-40 repeat protein); FGSG_05038: probable nuclear migration protein.

The two genes FGSG_05535 and FGSG_09988, annotated in GenRE as probable G protein alpha subunits, were shown to be dispensable for pathogenicity [36]. However, both proteins are connected to two seed proteins required for pathogenicity (reduced virulence phenotype). The seed proteins are: FGSG_09614 (GPA2) encoding a guanine nucleotide-binding protein alpha-3 subunit and FGSG_04104 (GPB1) encoding a guanine nucleotide-binding protein beta subunit. Both these seeds are involved in intracellular signalling. The two non-pathogenicity associated proteins as well as 7 others (white triangles in **Figure 5**) would all be predicted to be virulence associated proteins on the basis of having two links to pathogenicity associated seeds. This network neighbourhood contains mostly genes located in genomic regions with very low/no genetic recombination, which are also found in many other taxa. The only exception is FGSG_04618 which is located in a region of very high recombination towards the right hand end of chromosome 2, but which also has a wide taxon distribution. FGSG_09988 codes for the G protein alpha 3 subunit. This reveals the selective recruitment of the G protein alpha subunit to virulence signalling over the beta or gamma subunits in *F. graminearum*. Although this network analysis has revealed a multigene family to be associated with virulence, only through completion of the gene deletion experiments could the actual member recuited to virulence be revealed. None of other members of this cluster belong to multigene families. However the seven other predicted members of this G-protein cluster possess a WD repeat domain.

### Example 4: Prediction of Three Non Pathogenicity Associated Seeds as Potential Candidates for Virulence

The gene FGSG_00472 is connected to 5 seeds (**Figure S5**) and is annotated in GenRE as a probable cAMP dependent protein kinase. This gene has recently been shown to be required for pathogenicity and DON production *in planta* [26]. The 5 seed proteins in this cluster are all predicted to be protein kinases. In addition, in this cluster gene FGSG_00472 is connected to two additional potential candidates for virulence, namely genes FGSG_10095 and FGSG_01312. These genes are also annotated in GenRE as protein kinases and are themselves connected to either 3 or 4 protein kinase seeds. Both FGSG_10095 and FGSG_01312 have recently been shown to required for pathogenicity and DON production *in planta* [26]. Interestingly, the three newly verified virulence genes when deleted individually have only a minimal affects on *in vitro* growth, whereas all the seed genes in this cluster when deleted individually have a far greater affect on *in vitro* growth [26].

### Mapping of Recently Identified Kinase Proteins in *Fusarium graminearum* to the Integrated Network

The recent comprehensive study of the contribution of the predicted *F. graminearum* kinome to pathogenicity towards wheat ears, mycotoxin production and an additional 15 growth and development traits assessed *in vitro* [26] lead to the identification of 21 putative essential proteins, 44 proteins as having a proven role in disease formation (corresponding to reduced virulence) and 51 proteins with no apparent role in pathogenicity (refer to **Table S7**). We have used this data in an attempt to quantify the predictive accuracy of our combined network approach. Of these 44 new pathogenicity proteins, 23 correspond to predictions made within our integrated network (**Table 5**) and a further 4 are among our set of verified virulence seed proteins (FGSG_10313, FGSG_06385, FGSG_09903, FGSG_09897). In total, 11 of the essential for life proteins in [26] were among our predicted pathogenicity proteins as well as 22, which have been shown to be unaffected in virulence towards wheat ears. This latter figure highlights the problem with false positives. However, some of these single gene negative results may have occured via genetic redundancy, i. e. a member of a multigene family, where the role of the deleted gene can be fully taken over by the function of another related gene(s) and therefore no change in the phenotypic outcome is observed. Only by exploring the effects of deleting specific combinations of sequence related genes can these negative phenotypic effects be confirmed. A further possibility is that some of the predicted virulence genes may only be required for the infection of non-wheat host species.

**Table 5:** Comparison of the distribution of known phenotypes of the seeds within the four predicted networks.

Phenotype	Network type			
	Protein-protein interactions	Coexpression	Sequence similarity	Integrated
	Seeds			
**Reduced**	4	2	4	4
	**Predictions**			
**Essential**	5	0	9	11
**Reduced**	9	3	20	23
**Unaffected**	11	0	16	22

Counts of the different phenotypes according to the study by Wang and collegues [26] that were found among the predictions derived using four different networks.

Selecting only those predictions, which were made on the basis of slightly more stringent criteria, namely requiring at least 3 instead of 2 neighbours as seeds (of which there are 71) has only a small effect with lowering the number of correctly predicted proteins with the phenotype ‘reduced virulence’ to 21 and with phenotype ‘unaffected’ to 17.

### Chromosomal Location of the Predicted Pathogenicity Associated Proteins

When the newly sequenced *F. graminearum* genome of strain PH-1 and partial sequence information for a second strain GZ3639 were aligned to the available genetic map involving both these strains, this revealed an unanticipated result. Cuomo et al., (2007) described a genome, where the four *F. graminearum* chromosomes were unevenly divided into two types of genomic landscape. The majority of the genome exhibited minimal DNA polymorphism and a low rate of recombination between the two sequenced strains and the gene sequences predicted were also shared with two other Fusarium species, *F. oxysporum* and *F. verticillioides*. Separating these large blocks of conserved DNA, were several smaller regions with high DNA polymorphism, a very high recombination frequency, and these contained many of the predicted gene sequences considered to be unique to *F. graminearum*. These small unique regions of the genome were located in both the sub-telomeric and interstitial regions of each chromosome and were proposed to be the fusion sites of ancestral smaller chromosomes.

**Figure 6:**
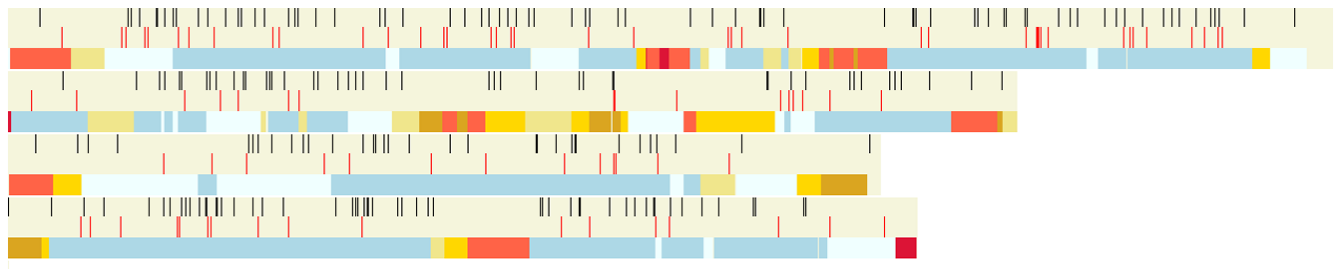
Position of the predictions in relation to the 4 chromosomes of *Fusarium graminearum*. The predicted virulence genes are shown as black vertical bars in track 1 for each chromosome. The verified virulence seeds (red bars) are depicted in track 2. Recombination frequency across the chromosomes is depicted in track 3 using a colour gradient (white (0.0) lowest to crimson (>8 cM highest). The various colours in track 3 for each chromosome indicate the frequence of recombination (cM/27 kb), i. e. # clBeige 1 clKhaki 2 clGold 3 clGoldenRod 4 clTomato 8 clCrimson. The numbers between the colours are boundary values in cM/27 kb. Beige represents the lowest and crimson the highest recombination frequency [47]. (Image generated using OmniMapFree [27]).

Due to the unusual topology of the *F. graminearum* genome landscape, the chromosomal positioning of the predicted virulence genes accross the four *Fusarium graminearum* chromosomes was explored (**Figure 6**). Visual inspection revealed that most of the virulence genes predictions lie in the lower recombination conserved part of the chromosomes (white and blue). However, four predicted virulence genes reside in chromosome regions with a high/very high recombination frequency (4 cM–8 cM, red and >8 cM crimson), namely – FGSG_00071 (**Figure 4**), FGSG_15983, FGSG_04618 (**Figure 5**) and FGSG_16412. Therefore the rarer type of genome landscape is explored in this network analysis. These 4 predicted virulence genes are found in many other species.

### The Predicted Virulence Associated Protein Set Shows an Increased Abundance in the Functional Categories Defense/Virulence and Cellular Communication

The functional classification system developed by the Munich Information Centre for Protein Sequences (MIPS) allows the automatic annotation of protein sets into 20 high level functional categories (Funcat) [37] (http://mips.helmholtz-muenchen.de/genre/proj/FGDB/). We hypothesised that successful prediction of virulence associated protein candidates using networks should also increase the annotation frequency of proteins belonging to Funcat groups comprising proteins involved in virulence and protein-protein interactions. Both the protein sets for the seeds and the predicted virulence associated proteins were compared (**Table 6**). A chi-square test showed that both groups are significantly different (P≥0.001). The Funcat groups 14 (protein fate), 30 (cellular communication/signal transduction mechanism) and 32 (cell rescue, defense and virulence) were increased, while the number of proteins belonging to Funcat group 99 (unclassified proteins and others) was strongly reduced.

**Table 6:** Funcat analysis of the verified virulence seeds and candidate virulence associated proteins.

The main functional categories		Seeds (%)	Candidates (%)
1	metabolism	9.9	7.6
2	energy	0.8	1.6
10	cell cycle and dna processing	4.3	7.1
11	transcription	9.1	4.4
12	protein synthesis	0.4	0.8
**14**	**protein fate (folding, modification, destination)**	**4.0**	**11.2**
16	protein with binding function or cofactor requirement	10.3	10.3
18	regulation of metabolism and protein function	4.0	4.4
20	cellular transport, transport facilities and transport routes	1.6	4.3
**30**	**cellular communication/signal transduction mechanism**	**4.0**	**9.3**
**32**	**cell rescue, defense and virulence**	**1.6**	**4.5**
34	interaction with the environment	3.2	3.7
36	systemic interaction with the environment	0.8	1.2
38	transposable elements, viral and plasmid proteins	0.0	0.1
40	cell fate	4.3	2.6
41	development	2.0	1.2
42	biogenesis of cellular components	3.2	4.4
43	cell type differentiation	6.7	4.2
45	tissue differentiation	0.4	0.3
47	organ differentiation	0.4	0.5
70	Subcellular localization	9.5	9.4
**99**	**unclassified proteins and others**	**19.8** &	**6.9**

& recovered from forward genetic screens.

## Discussion

The integration of multiple types of data such as co-expression, protein-protein interaction and sequence relatedness can provide biological context to particular proteins by showing their relationship to other proteins. In some cases such an approach can provide enhanced annotation or indeed the first annotation for a sequence. For example a protein of unknown function may be strongly co-expressed or may interact with a number of proteins whose functions are known and this may help in narrowing down the possible roles of the previously unannotated protein. Here we used a similar ‘guilt by association’ approach to examine the network neighbourhood of proteins known to be involved in pathogenicity or virulence for the fungal Ascomycete species *Fusarium graminearium*. There is a large amount of biological, genome and transcriptome information publically available for this species and other pathogenic *Fusarium* species [4], [38]–[40] because of the ever rising economic global importance of Fusarium ear blight disease (www.scabusa.org, [2], [41].

This study greatly extends the previous network study of ([12] - Liu et al.). The integrated relationship network developed in this study leads to 215 predictions, of which 29 are hypothetical proteins (as annotated by the Fusarium Database ([6] - Wong et al.) and 25 are fungal specific. The integrated network was particularly informative and predicted 15 proteins linked to virulence that were only revealed in this network. Of these, FGSG_06878 has now been linked to virulence via the shotgun functional analysis of the predicted kinome ([21]-Wang et al.), whilst FGSG_03535 (TRI4) is known to be highly upregulated *in planta* and is required for the synthesis of of the DON mycotoxin. The function of the other 13 predicted virulence associated proteins from the integrated network has not yet been established (1) and/or tested (12). In addition, this study generated four predictions, where the prediction was linked to either 7 or 8 seeds. Of these FGSG_00071 (TRI1), FGSG_07251 and FGSG_10066 have each recently been shown to be required for virulence, whilst the FGSG_09715 single gene deletion mutant was unaffected in pathogenicity towards wheat floral tissue. This level of correct prediction amongst the sequences most highly connected to the verified virulence seeds could be a way of further prioritising the list.

Amongst the 215 predictions, several proteins are considered to have a direct role in virulence because these are required for the production of the DON mycotoxin virulence factor, i.e. example 2. However, the rest of the predictions could play either a direct or indirect role in virulence. The analysis of the sequence type and protein size distribution of the predictions would indicate that this study has underexplored the possible effector component of *Fusarium graminearum.* At the present time we consider most of the predicted virulence associated proteins identified in this study to have an indirect role in virulence and could be seen as system components [18].

One of the caveats with the approach we have taken is that predictions can be biased in favour of nodes with high degree centrality values. The degree centrality of a node in the network is a measure of the number of edges connected to that node, and the higher the value the more ‘hub-like’ is the node. We used the Kolmogorov-Smirnov test (see, for example, [42]) to compare the (cumulative) distributions of each of the three possible pairs of degree centrality data sets, namely (i) the nodes corresponding to the verified virulence seeds vs. the nodes of the integrated network, (ii) the nodes corresponding to the predicted virulence associated proteins vs. the nodes of the integrated network and (iii) the nodes corresponding to the predicted virulence associated proteins vs. the nodes corresponding to the verified virulence seeds. The test revealed that there was no significant difference for (i) but that there was a highly significant difference for (ii) and (iii). This may reflect a bias in the predictions towards high degree centrality nodes, as such nodes are more likely to be connected to two or more seed proteins.

Another potential limitation of the approach is that for many pathogens (excluding well studied examples such as *Fusarium graminearum* (see for example [7]), *Magnaporthe oryzae*, a rice pathogen and *Ustilago maydis,* a maize pathogen, there is typically very limited information on proteins that have been investigated experimentally for their contribution to virulence and that can act as seeds. Additionally the set of verified virulence seeds is most likely biased with certain types of protein being the subject of more intense biological investigations. For example, for *F. graminearum* although the analysis of the predicted transcription factors and protein kinases (the kinome) has been thoroughly explored ([43] [26] so far the function of the predicted secretome has not [44]. This means that currently the network space is not evenly sampled and may result in many potential targets being missed. Over the next few years this problem could either become worse if the community focusses on genes and gene families already known to essential for infection and/or disease formation in other pathogenic species, or the position may improve as the results from large forward genetic screens for pathogenicity factors and/or via the screening of hypothetical and conserved hypothetical sequences occurs.

Recently, a large scale targeted gene disruption study to search for novel secreted fungal virulence genes was reported for the rice blast pathogen *Magnaporthe orzyae* [45]. In total, 78 putative secreted proteins, most with low sequences similarlity, but highly expressed during the early stages of plant infection, were tested for function. Only one *M. oryzae* gene was shown to be required for virulence in cereal plants. Deletion of the orthologous gene reduced the virulence of another fungal pathogen *Colletotrichum orbiculaire*, which causes anthracnose disease on non-cereal plants. This novel virulence gene has a very restricted fungal taxon distribution. Overall, this recent large experimental biology study reveals just how low a level of predictive success was achieved (1.28%) from an initial highly focussed bioinformatics analyses. Therefore at the present time, the sensitivity of our predictions for *F. graminearum* virulence associated proteins from using the integrated network (1.66%, **Table S4**) is comparable to that achieved using a partially bioinformatically guided, direct experimental approach.

Once genome sequence and gene function information is published on different strains of the same species, several closely related species, or *formae specialis,* then the power of this type of predictive technique is likely to greatly increase. For example, within the Fusaria the number of species under experimental investigation is gradually expanding and involves the use of a range of cereal, non-cereal and mammalian host infecting species. These studies include *F. oxysporum* f.sp *lycopersici* and various other *formae specialis,* which infect different dicotyledonous plant species, *F. solani* as well as *F. verticillioides*, *F. culmorum* and *F. pseudograminearum,* which infect a range of cereal hosts. Also, it is anticipated that in the next five years due to the increased efficiency of generating single gene deletion strains in specific plant pathogenic species, this type of integrated network could be used for comparative analyses involving evolutionarily closely related fungal species with subtly different infection routes and/or host ranges.

The protein interaction component of the integrated network representing predicted interactions [14] was built using known interaction data from 7 non-pathogenic, non-filamentous fungal organisms using information from interologs and domain-domain interactions. Therefore interactions between *Fusarium* specific proteins will not have been captured. The identification within the integrated network of a prediction involved in trichothecene mycotoxin production (**Figure 2**), indicates the value of including co-expresssion data. With the increasing use of next generation sequencing technologies to explore the interaction transcriptome in greater detail, it is conceivable that co-expression information on different phases of the interaction could be used to further refine the virulence associated protein predictions.

Exploration of the network together with expert biological knowledge about the predicted proteins in the neighbourhoods of verified virulence proteins may lead to a further reduction in hypothesis space and prioritisation to a few genes that could be the target for experimental investigation. However, two separate *F. graminearum* large studies recently published, explored the function of the 709 predicted transcription factors (TAPs) [43] and the 116 predicted protein kinases [26], indicate that the testing of the entire 215 predictions in a focussed project would be feasible via a consortium research approach.

## Methods

### The Integrated Network

Starting with version 3.3 of PHI-base, *Fusarium graminearum* genes were selected whose contributions to virulence have been tested experimentally and were classified according to whether they have an effect or not. Further expert curation of more recent literature for this study added more *Fusarium graminearum* genes that experiments suggest are involved in virulence and that are currently not in PHI-base Vers. 3.3. **Table S1** shows the complete list of seed genes. In total, these 133 experimentally-tested genes are referred to as the verified virulence (VV) ‘seed’ genes. The mapping of *Fusarium graminearum* entries in PHI-base to corresponding sequences taken from the latest annotation of the *Fusarium graminearum* genome at the Broad Institute (gene call FG3) was carried out using BLAST and manually reviewed. The total numbers of VV seeds is 100, and the ‘virulence unaffected’ seeds is 33. The *F. graminearum* genome is predicted to code for 13,332 proteins.

We have described the construction of the integrated network for *Fusarium graminearum* and explored its community structure in [19]. The network was constructed using information from three component data sources, namely gene co-expression, protein sequence similarity and predicted protein-protein interactions. The co-expression component of the network was constructed from the complete publically-available set (12 experiments, 158 individual slides) of *Fusarium* expression studies form PLEXdb [15] that used *Fusarium* Affymetrix GeneChip array [5]. This included 6 *in planta* experiments and 6 *in vitro* studies using the wild-type sequenced PH-1 strain and/or single gene deletion mutants generated in the PH-1 strain on which the GeneChip array was designed (**Table S7**). The data was downloaded in the form of.CEL files, pooled and normalised using the Robust Multichip Average (Irizarry et al., 2003), at which point a data matrix of size 18069 (genes) X 158 (samples) was constructed. The similarity of expression profiles was measured using weighted Pearson correlation coefficient, according to the method of [46]. The sparse network was constructed from the correlation matrix by applying a threshold of 0.88. This value was determined to be optimal for this dataset using the method of Elo et al. (2007), which derives the optimal correlation cut-off value based on the topological properties of the network. The probe set IDs from the FG3 annotation of *Fusarium* [6] were integrated using a mapping file obtained from MIPS (http://mips.helmholtz-muenchen.de/genre/proj/FGDB/). The sequence similarity network was constructed from the results of an all-versus-all sequence matching of the proteins in version 3.2 of the Fusarium annotation at (http://mips.helmholtz-muenchen.de/genre/proj/FGDB/) implemented on a TimeLogic® Tera-BLAST™ (Active Motif Inc., Carlsbad, CA). The network was constructed by creating a “similar sequence” edge joining the two nodes (genes) when there was a pairwise similarity observed between their sequences (bidirectional hit) with expected value of less than 10^−6^. The co-expression network, the predicted core PPI of Zhao et al [14], the sequence similarity network and the mutant phenotype annotations (from PHI-base and the more recently curated literature) were imported into the Ondex data integration and visualisation system [16] (www.ondex.org) and combined. Merging the nodes that had the same gene accession resulted in the union of the two networks. The coexpression values and scores derived from BLAST were included as weights on appropriate edges and are included in the final integrated network available with this paper. The explanation about how the BLAST scores were calculated and the distribution of these values for all edges used in predictions are included as a **Figure S6**. It is, therefore possible to adjust the threshold further in Ondex network visualisation software and explore what effects it would have on the network and the predictions.

In this study we were interested in the potential of the network for prediction. The prediction of virulence genes was achieved by implementing a new plug-in software module for the Ondex system. The plug-in works by creating a set of sub-graphs that include genes annotated to be of relevance to virulence (the verified virulence seeds) and their nearest neighbours with respect to co-expression, PPI and sequence similarity in the constituent and combined networks. The genes were predicted to be likely important for virulence if there were at least two known virulence-relevant genes found in their immediate network neighbourhood, in a similar manner to that of Liu et al [12]. The seed nodes, the predictions and the edges connecting predictions to seeds were “tagged” to create gene lists, which could then be used to select relevant subsets of the network for visualisation in the graphical user interface of Ondex.

The Ondex software can be downloaded from www.ondex.org. The integrated network, seed genes and predictions are made available in **File S1**.

## Supporting Information

Figure S1 The entire integrated network containing the predicted virulence associated gene FGSG_06878 connected to 8 verified virulence seeds.
(DOCX)

Figure S2 The local integrated network containing the predicted virulence associated gene 07251 connected to 7 verified virulence seeds.
(DOCX)

Figure S3 The integrated network containing the predicted virulence associated gene FGSG_09715 connected to 7 verified virulence seeds.
(DOCX)

Figure S4 The integrated network containing the predicted virulence associated gene FGSG_10066 connected to 7 verified virulence seeds.
(DOCX)

Figure S5 The integrated network containing the predicted virulence associated gene FGSG_00472 connected to 5 verified virulence seeds.
(DOCX)

Figure S6 The distribution of e-values for sequence similarity edges that were used for deriving predictions.
(DOCX)

Table S1 List of 133 seed verified virulence (VV) genes.
(DOCX)

Table S2 Selected annotation for the 215 predicted virulence associated proteins.
(XLSX)

Table S3 The ratios of seed associated to all other edges for all of the proteins predicted to be associated with virulence.
(DOCX)

Table S4 Estimating the predictive power of the four different networks.
(DOCX)

Table S5 Heatmap showing the taxonomic diversity of the matches to the 215 predictions.
(DOCX)

Table S6 Prediction of FGSG_09715, FGSG_07251 and FGSG_10066 as virulence associated proteins.
(DOCX)

Table S7 Mapping the data from Wang et al to the integrated network.
(DOCX)

Table S8 The publically available *F. graminearum* microarray gene expression datasets used in this study.
(DOCX)

File S1 ZIP archive file for Ondex containing the integrated network, seed genes and predictions in OXL format.
(ZIP)
