# Supplementary material for: Network-Based Data Integration for Selecting Candidate Virulence Associated Proteins in the Cereal Infecting Fungus Fusarium graminearum
Source: PLoS One. 2013 Jul 4;8(7):e67926. doi: 10.1371/journal.pone.0067926 (PMC3701590; doi:10.1371/journal.pone.0067926)
Supplement: Figure S1 — The entire integrated network containing the predicted virulence associated gene FGSG_06878 connected to 8 verified virulence seeds. (DOCX) [file pone.0067926.s001.docx]

**Figure S1: The integrated network containing the predicted virulence gene FGSG_06878 connected to 8 verified virulence seeds (nodes). FGSG_06878 is one of the main hubs (mid left) and is part of the three distinct modules.** The various node colours of the seeds as shown in the legend indicate the experimentally determined outcomes. The magenta coloured edges predicted PPI information, blue edges predicted co-expression information and the green coloured edges predict sequence similarity information. There are many additional virulence predictions in this neighbourhood (small white triangles).

**
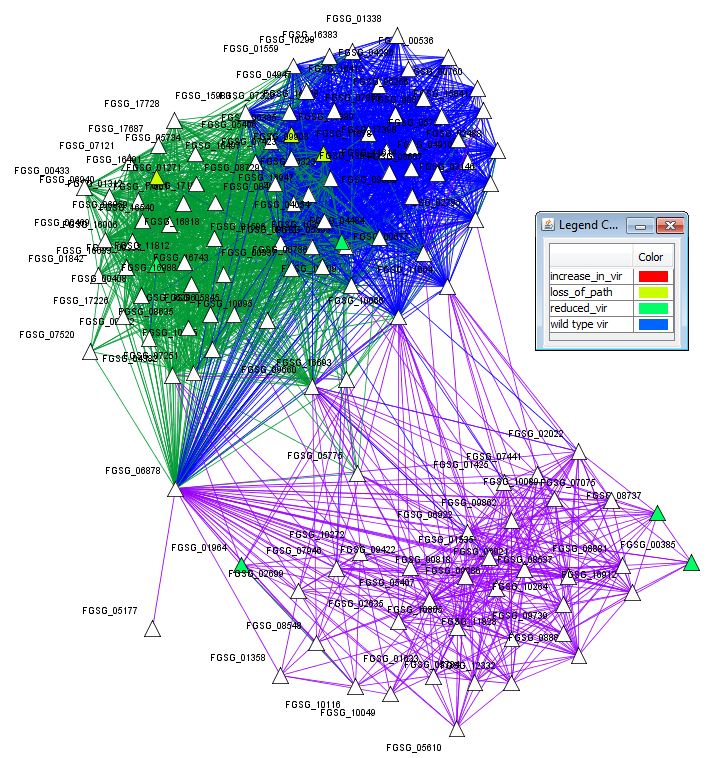
**
