# Supplementary material for: Network-Based Data Integration for Selecting Candidate Virulence Associated Proteins in the Cereal Infecting Fungus Fusarium graminearum
Source: PLoS One. 2013 Jul 4;8(7):e67926. doi: 10.1371/journal.pone.0067926 (PMC3701590; doi:10.1371/journal.pone.0067926)

**Figure S2: The integrated network containing the predicted virulence gene FGSG_06878 connected to 7 verified virulence seeds (nodes).** See Supplementary Figure 1 and legend for a description of the colour codes used for the node and edges


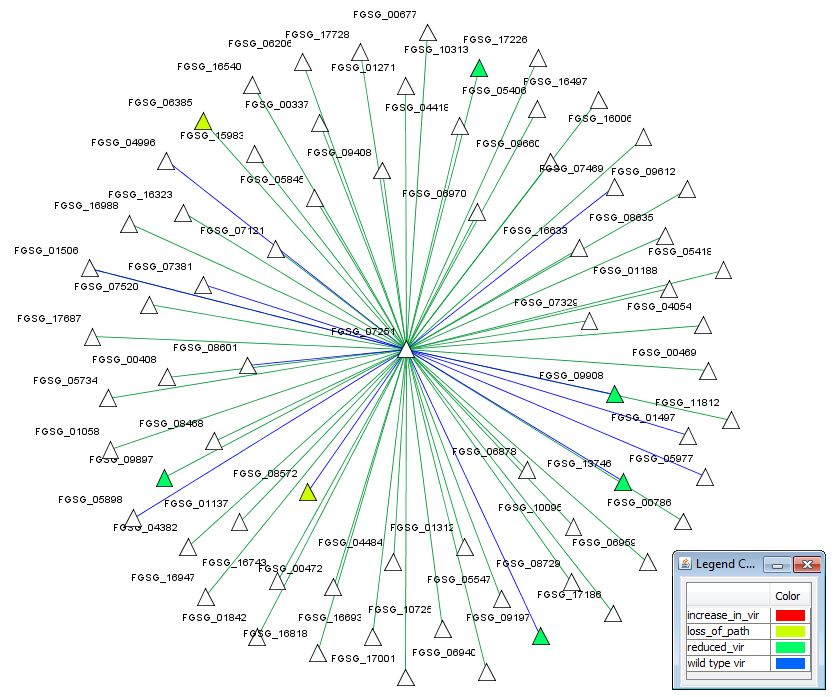

Supplement: Figure S2 — The local integrated network containing the predicted virulence associated gene 07251 connected to 7 verified virulence seeds. (DOCX) [file pone.0067926.s002.docx]
