# Supplementary material for: Network-Based Data Integration for Selecting Candidate Virulence Associated Proteins in the Cereal Infecting Fungus Fusarium graminearum
Source: PLoS One. 2013 Jul 4;8(7):e67926. doi: 10.1371/journal.pone.0067926 (PMC3701590; doi:10.1371/journal.pone.0067926)
Supplement: Figure S6 — The distribution of e-values for sequence similarity edges that were used for deriving predictions. (DOCX) [file pone.0067926.s006.docx]

**Figure S6. The distribution of e-values for sequence similarity edges that were used for deriving predictions.**


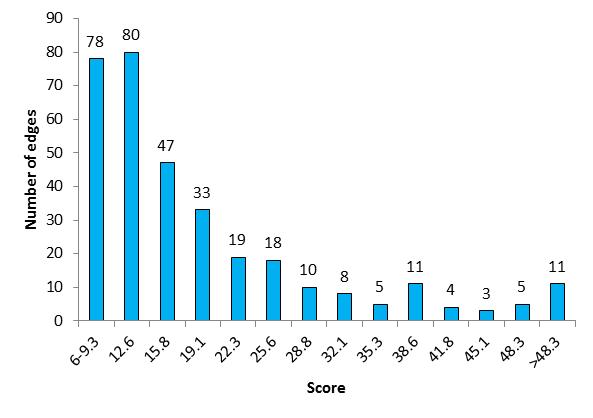


The score was derived by taking a negative logarithm of an actual e-value, therefore the numbers shown correspond to the exponents of the original e-values (e.g. a score of 1e-6 would become 6). As only bi-directional BLAST hits were included, the scores of hits in both directions were averaged to give the score used for this chart.
