# Supplementary material for: Network-Based Data Integration for Selecting Candidate Virulence Associated Proteins in the Cereal Infecting Fungus Fusarium graminearum
Source: PLoS One. 2013 Jul 4;8(7):e67926. doi: 10.1371/journal.pone.0067926 (PMC3701590; doi:10.1371/journal.pone.0067926)
Supplement: Table S1 — List of 133 seed verified virulence (VV) genes. (DOCX) [file pone.0067926.s007.docx]

**Supplemental Table S1:** List of 133 seed verified virulence (VV) genes.

The seed verified virulence (VV) genes ordered by FGSG identifier, the gene name, the corresponding PHI-base identifier (where the genes are in PHI-base, version 32), and the associated phenotype. The last column shows changes in the identifiers made in the recent gene call (v32) of FGDB.

|  | **FGSG number** | **Gen Name** | **PHIbase ID** | **Phenotype** | **V32 changes** |
| --- | --- | --- | --- | --- | --- |
| 1 | FGSG_00007 |  |  | increase_in_vir | FGSG_17598 |
| 2 | FGSG_00147 | GzZC305 |  | reduced_vir |  |
| 3 | FGSG_00324 | GzMyb002 |  | reduced_vir |  |
| 4 | FGSG_00332 | TBL1 | PHI:446 | reduced_vir |  |
| 5 | FGSG_00376 | NOS1 | PHI:445 | reduced_vir |  |
| 6 | FGSG_00385 | GzC2HMG002 |  | reduced_vir |  |
| 7 | FGSG_00477 | GzC2H003 |  | reduced_vir |  |
| 8 | FGSG_00515 | GzbZIP001 |  | reduced_vir |  |
| 9 | FGSG_00574 | GzZC302 |  | reduced_vir |  |
| 10 | FGSG_00719 | GzZC282 |  | reduced_vir |  |
| 11 | FGSG_00729 | GzC2HMG005 |  | reduced_vir |  |
| 12 | FGSG_00950 | SYN1 |  | reduced_vir |  |
| 13 | FGSG_01022 | GzC2H007 |  | reduced_vir |  |
| 14 | FGSG_01106 | GzC2H008 |  | reduced_vir |  |
| 15 | FGSG_01176 | GzZC248 |  | reduced_vir |  |
| 16 | FGSG_01182 | GzCCAAT002 |  | reduced_vir |  |
| 17 | FGSG_01293 | GzZC236 |  | reduced_vir |  |
| 18 | FGSG_01307 | GzbHLH005 |  | reduced_vir |  |
| 19 | FGSG_01341 | GzC2H013 |  | reduced_vir |  |
| 20 | FGSG_01350 | GzC2H014 |  | reduced_vir |  |
| 21 | FGSG_01364 | CCH1 | PHI:1080 | wild type vir |  |
| 22 | FGSG_01555 | ZIF1 | PHI:444 | reduced_vir |  |
| 23 | FGSG_01665 | FSR1 | PHI:731 | reduced_vir |  |
| 24 | FGSG_01790 | PKS11 |  | wild type vir |  |
| 25 | FGSG_01932 | CBL1 | PHI:443 | reduced_vir |  |
| 26 | FGSG_01939 | ARG2 | PHI:743 | reduced_vir |  |
| 27 | FGSG_01964 | CHS5 |  | reduced_vir |  |
| 28 | FGSG_02095 | FBP1 | PHI:733 | reduced_vir |  |
| 29 | FGSG_02324 | AUR1 | PHI:720 | wild type vir |  |
| 30 | FGSG_02328 | GIP1 |  | wild type vir |  |
| 31 | FGSG_02395 | PKS13 | PHI:713 | wild type vir | FGSG_15980 PKS13 |
| 32 | FGSG_02398 | ZEB2 | PHI:715 | wild type vir |  |
| 33 | FGSG_02506 | ADE5 | PHI:744 | reduced_vir |  |
| 34 | FGSG_02527 | GzDDT |  | reduced_vir |  |
| 35 | FGSG_03340 | PKS17 |  | wild type vir |  |
| 36 | FGSG_03536 | TRI6 | PHI:439 | reduced_vir | FGSG_16251 (TRI 6) |
| 37 | FGSG_03538 | TRI10 |  | reduced_vir |  |
| 38 | FGSG_03543 | TRI14 | PHI:525 | reduced_vir |  |
| 39 | FGSG_03747 | NPS6 | PHI:1007 | reduced_vir |  |
| 40 | FGSG_03964 | GRS1 |  | wild type vir |  |
| 41 | FGSG_04083 | GzC2H024 |  | reduced_vir |  |
| 42 | FGSG_04104 | GPB1 |  | reduced_vir |  |
| 43 | FGSG_04111 | PTC1 |  | reduced_vir |  |
| 44 | FGSG_04134 | GzC2H026 |  | loss_of_path |  |
| 45 | FGSG_04220 | GzAPSES001 |  | reduced_vir |  |
| 46 | FGSG_04355 | CID1 |  | reduced_vir |  |
| 47 | FGSG_04488 | PLSP1 | PHI:717 | wild type vir |  |
| 48 | FGSG_04510 |  | PHI:1087 | wild type vir |  |
| 49 | FGSG_04610 |  | PHI:1094 | wild type vir |  |
| 50 | FGSG_04694 | PKS2 |  | wild type vir |  |
| 51 | FGSG_05171 | GzbZIP007 |  | reduced_vir |  |
| 52 | FGSG_05304 | GzCCAAT004 |  | reduced_vir |  |
| 53 | FGSG_05371 | SID1 |  | wild type vir |  |
| 54 | FGSG_05388 | FgFSR1 |  | loss_of_path |  |
| 55 | FGSG_05484 | STE11 | PHI:1016 | loss_of_path | FGSG_16491 FST11 |
| 56 | FGSG_05535 | GPA1 |  | wild type vir |  |
| 57 | FGSG_05593 | MT2 |  | reduced_vir |  |
| 58 | FGSG_05658 | GzmetE | PHI:355 | reduced_vir |  |
| 59 | FGSG_05794 | PKS5 |  | wild type vir | FGSG_16550,  FGSG_17677 Fused FGSG_05794 |
| 60 | FGSG_05906 | FGL1 | PHI:432 | reduced_vir |  |
| 61 | FGSG_06071 | GzAT001 |  | loss_of_path |  |
| 62 | FGSG_06228 | GzWing013 |  | reduced_vir | FGSG_16620 |
| 63 | FGSG_06291 | GzBrom002 |  | loss_of_path |  |
| 64 | FGSG_06385 | MAP1(gpmk1) | PHI:309 | loss_of_path |  |
| 65 | FGSG_06427 | GzC2H042 |  | reduced_vir |  |
| 66 | FGSG_06631 | CPS1 | PHI:304 | reduced_vir |  |
| 67 | FGSG_06651 | GzbZIP010 |  | reduced_vir |  |
| 68 | FGSG_06680 | MES1 | PHI:1078 | reduced_vir | FGSG_16701 |
| 69 | FGSG_06871 | GzC2H045 |  | reduced_vir |  |
| 70 | FGSG_06874 | TOP1 |  | reduced_vir |  |
| 71 | FGSG_06944 | GzWing015 |  | reduced_vir |  |
| 72 | FGSG_06948 | Gzscp |  | loss_of_path |  |
| 73 | FGSG_07062 |  | PHI:1096 | wild type vir |  |
| 74 | FGSG_07067 | GzZC232 |  | reduced_vir |  |
| 75 | FGSG_07133 | GzZC230 |  | reduced_vir |  |
| 76 | FGSG_07226 | KSA1 |  | wild type vir |  |
| 77 | FGSG_07798 | FUS1 |  | wild type vir |  |
| 78 | FGSG_07928 | GzC2H059 |  | reduced_vir |  |
| 79 | FGSG_08028 | GzZC120 |  | reduced_vir |  |
| 80 | FGSG_08182 | GzZC116 |  | reduced_vir |  |
| 81 | FGSG_08208 | PKS6 |  | wild type vir |  |
| 82 | FGSG_08481 | GzWing018 |  | reduced_vir |  |
| 83 | FGSG_08572 | GzWing019 |  | loss_of_path |  |
| 84 | FGSG_08617 | GzC2H066 |  | reduced_vir |  |
| 85 | FGSG_08695 | PLS1 | PHI:1079 | wild type vir |  |
| 86 | FGSG_08719 | GzWing020 |  | loss_of_path | FGSG_16982 |
| 87 | FGSG_08737 | GzOB031 |  | reduced_vir |  |
| 88 | FGSG_08769 | GzZC108 |  | reduced_vir |  |
| 89 | FGSG_08795 | PKS7 |  | wild type vir |  |
| 90 | FGSG_09019 | GzHOME009 |  | reduced_vir |  |
| 91 | FGSG_09182 | PGL1 |  | wild type vir | FGSG_17168 PKS3 |
| 92 | FGSG_09197 | HMR1 | PHI:1006 | reduced_vir |  |
| 93 | FGSG_09339 | GzMADS003 |  | reduced_vir |  |
| 94 | FGSG_09614 | GPA2 |  | reduced_vir |  |
| 95 | FGSG_09654 | GzOB038 |  | reduced_vir |  |
| 96 | FGSG_09759 |  | PHI:1088 | wild type vir |  |
| 97 | FGSG_09832 | GzbZIP016 |  | reduced_vir |  |
| 98 | FGSG_09868 | GzHMG029 |  | reduced_vir |  |
| 99 | FGSG_09891 | AB1 |  | wild type vir |  |
| 100 | FGSG_09893 | AB2 |  | wild type vir |  |
| 101 | FGSG_09895 | NTH1 |  | reduced_vir |  |
| 102 | FGSG_09896 | ICL1 |  | wild type vir |  |
| 103 | FGSG_09897 | SNF1 |  | reduced_vir |  |
| 104 | FGSG_09900 | AB3 |  | wild type vir | FGSG_17290 |
| 105 | FGSG_09903 | STE7 | PHI:1004 | loss_of_path |  |
| 106 | FGSG_09905 | AB4 |  | wild type vir |  |
| 107 | FGSG_09906 | AB4 |  | wild type vir |  |
| 108 | FGSG_09907 | FCV1 |  | reduced_vir |  |
| 109 | FGSG_09908 | PKAR |  | reduced_vir |  |
| 110 | FGSG_09928 | SYN2 |  | reduced_vir |  |
| 111 | FGSG_09988 | GPA3 |  | wild type vir |  |
| 112 | FGSG_09992 | GzNH001 |  | loss_of_path | FGSG_17300 |
| 113 | FGSG_10114 | RAS2 | PHI:861 | reduced_vir |  |
| 114 | FGSG_10129 | FgStuA |  | reduced_vir |  |
| 115 | FGSG_10142 | GzbZIP017 |  | reduced_vir |  |
| 116 | FGSG_10179 | GzLam002 |  | reduced_vir |  |
| 117 | FGSG_10313 | MGV1 | PHI:266 | reduced_vir |  |
| 118 | FGSG_10384 | GzAPSES004 |  | reduced_vir |  |
| 119 | FGSG_10397 | FGSG_10397 |  | increase_in_vir |  |
| 120 | FGSG_10464 | PKS9 |  | wild type vir |  |
| 121 | FGSG_10517 | GzC2H090 |  | reduced_vir | FGSG_17381 |
| 122 | FGSG_10548 | PKS1 |  | wild type vir | FGSG_17387 PKS1 |
| 123 | FGSG_10716 | GzCCHC011 |  | loss_of_path |  |
| 124 | FGSG_10825 | MSY1 | PHI:442 | reduced_vir |  |
| 125 | FGSG_11025 | TRI15 |  | increase_in_vir |  |
| 126 | FGSG_11416 | GzC2H093 |  | reduced_vir | FGSG_17555,  FGSG_17556 Fused |
| 127 | FGSG_12039 | CHS7 | PHI:337 | reduced_vir | FGSG_15914 |
| 128 | FGSG_12126 | PKS4 | PHI:714 | wild type vir | FGSG_17745 PKS4 |
| 129 | FGSG_12781 | GzMyb017 |  | loss_of_path | FGSG_16521 |
| 130 | FGSG_12977 | GzZC087 |  | loss_of_path |  |
| 131 | FGSG_13120 | GzOB047 |  | reduced_vir |  |
| 132 | FGSG_13711 | GzC2H105 |  | reduced_vir | FGSG_17323 Split FGSG_13710,  FGSG_13711 |
| 133 | FGSG_13746 | GzNot002 |  | reduced_vir |  |
