# Supplementary material for: Network-Based Data Integration for Selecting Candidate Virulence Associated Proteins in the Cereal Infecting Fungus Fusarium graminearum
Source: PLoS One. 2013 Jul 4;8(7):e67926. doi: 10.1371/journal.pone.0067926 (PMC3701590; doi:10.1371/journal.pone.0067926)
Supplement: Table S3 — The ratios of seed associated to all other edges for all of the proteins predicted to be associated with virulence. (DOCX) [file pone.0067926.s009.docx]

**Table S3: The ratios of seed associated to all other edges for all of the proteins predicted to be associated with virulence.** This table shows the ratio of edges connected to seeds to the total number of edges for all of the predictions reported in this study. Columns two to four give the ratios for the individual networks. The fifth column shows the ratio if all types of edges were considered simultaneously. The last column lists all the seeds connected to that prediction in the combined network. The values are sorted in descending order with respect to edge ratio in the integrated network.

| **FGDG id** | **Protein-protein interaction** | **Co-expression** | **Sequence similarity** | **Integrated** | **Seeds** |
| --- | --- | --- | --- | --- | --- |
| **FGSG_01548** | 0.00 | 1.00 | 0.00 | 1.00 | FGSG_09928 FGSG_15914 |
| **FGSG_01949** | 0.00 | 0.00 | 1.00 | 0.67 | FGSG_01964 FGSG_15914 |
| **FGSG_04293** | 0.00 | 0.00 | 0.50 | 0.50 | FGSG_01022 FGSG_01350 |
| **FGSG_16816** | 0.00 | 0.00 | 0.45 | 0.45 | FGSG_01022 FGSG_01350 FGSG_07928 FGSG_08617 FGSG_01341 |
| **FGSG_01228** | 0.00 | 0.40 | 0.00 | 0.40 | FGSG_00385 FGSG_08737 |
| **FGSG_16768** | 0.00 | 0.00 | 0.40 | 0.40 | FGSG_01022 FGSG_01350 FGSG_08617 FGSG_01341 |
| **FGSG_00460** | 0.67 | 0.00 | 0.00 | 0.40 | FGSG_06385 FGSG_04355 |
| **FGSG_03534** | 0.00 | 0.38 | 0.00 | 0.38 | FGSG_10397 FGSG_17598 FGSG_03543 |
| **FGSG_16967** | 0.00 | 0.00 | 0.38 | 0.38 | FGSG_07928 FGSG_06871 FGSG_08617 |
| **FGSG_00342** | 0.00 | 0.00 | 0.33 | 0.33 | FGSG_01022 FGSG_08617 |
| **FGSG_10470** | 0.00 | 0.00 | 0.33 | 0.33 | FGSG_01350 FGSG_01341 |
| **FGSG_17038** | 0.00 | 0.00 | 0.33 | 0.33 | FGSG_01350 FGSG_06871 FGSG_08617 FGSG_01341 |
| **FGSG_00792** | 0.00 | 0.00 | 0.29 | 0.29 | FGSG_16491 FGSG_09897 |
| **FGSG_13314** | 0.00 | 0.00 | 0.29 | 0.29 | FGSG_01350 FGSG_06871 |
| **FGSG_09410** | 0.00 | 0.00 | 0.27 | 0.27 | FGSG_01022 FGSG_08617 FGSG_01341 |
| **FGSG_16005** | 0.00 | 0.00 | 0.33 | 0.25 | FGSG_01964 FGSG_15914 |
| **FGSG_09715** | 0.00 | 0.00 | 0.38 | 0.23 | FGSG_01022 FGSG_01350 FGSG_07928 FGSG_08617 FGSG_06871 FGSG_06071 FGSG_01341 |
| **FGSG_10167** | 0.00 | 0.27 | 0.00 | 0.22 | FGSG_09928 FGSG_01555 FGSG_01964 FGSG_06071 |
| **FGSG_16273** | 0.00 | 0.00 | 0.33 | 0.22 | FGSG_01964 FGSG_15914 |
| **FGSG_15867** | 0.00 | 0.00 | 0.25 | 0.22 | FGSG_16491 FGSG_09897 |
| **FGSG_06550** | 0.00 | 0.00 | 0.29 | 0.22 | FGSG_01964 FGSG_15914 |
| **FGSG_06311** | 0.00 | 0.00 | 0.22 | 0.22 | FGSG_01350 FGSG_01341 |
| **FGSG_10822** | 0.23 | 0.00 | 0.00 | 0.21 | FGSG_10313 FGSG_06385 FGSG_06948 |
| **FGSG_06684** | 0.00 | 0.00 | 0.23 | 0.21 | FGSG_01022 FGSG_01350 FGSG_08617 |
| **FGSG_07310** | 0.00 | 0.00 | 0.29 | 0.21 | FGSG_01022 FGSG_01350 FGSG_06871 FGSG_01341 |
| **FGSG_07469** | 0.00 | 0.00 | 0.67 | 0.20 | FGSG_01964 FGSG_16982 FGSG_04111 |
| **FGSG_02743** | 0.00 | 0.00 | 0.19 | 0.19 | FGSG_01350 FGSG_06871 FGSG_00477 |
| **FGSG_09988** | 0.20 | 0.00 | 0.00 | 0.18 | FGSG_09614 FGSG_04104 |
| **FGSG_07520** | 0.00 | 0.00 | 0.18 | 0.18 | FGSG_10313 FGSG_16491 |
| **FGSG_05512** | 0.13 | 0.00 | 0.00 | 0.18 | FGSG_01939 FGSG_01106 FGSG_04355 |
| **FGSG_17028** | 0.18 | 0.00 | 0.00 | 0.18 | FGSG_06291 FGSG_04355 FGSG_13746 |
| **FGSG_01392** | 0.15 | 0.00 | 0.00 | 0.15 | FGSG_10313 FGSG_06948 |
| **FGSG_15757** | 0.00 | 0.16 | 0.00 | 0.14 | FGSG_01555 FGSG_06071 FGSG_15914 |
| **FGSG_00883** | 0.00 | 0.22 | 0.00 | 0.14 | FGSG_01106 FGSG_06071 |
| **FGSG_04053** | 0.00 | 0.00 | 0.14 | 0.14 | FGSG_10313 FGSG_06385 FGSG_09897 |
| **FGSG_07504** | 0.00 | 0.00 | 0.14 | 0.14 | FGSG_01022 FGSG_01341 |
| **FGSG_01410** | 0.00 | 0.00 | 0.67 | 0.14 | FGSG_01964 FGSG_16982 |
| **FGSG_09535** | 0.14 | 0.00 | 0.00 | 0.14 | FGSG_16620 FGSG_10313 |
| **FGSG_08691** | 0.00 | 0.00 | 0.12 | 0.14 | FGSG_06385 FGSG_16491 FGSG_09903 FGSG_10142 |
| **FGSG_16693** | 0.00 | 0.17 | 0.12 | 0.13 | FGSG_10313 FGSG_06385 FGSG_01964 FGSG_06071 |
| **FGSG_10808** | 0.00 | 0.13 | 0.00 | 0.13 | FGSG_01964 FGSG_15914 |
| **FGSG_02010** | 0.40 | 0.00 | 0.00 | 0.13 | FGSG_06291 FGSG_13746 |
| **FGSG_04418** | 0.00 | 0.00 | 0.13 | 0.13 | FGSG_10313 FGSG_06385 FGSG_16491 FGSG_09897 |
| **FGSG_17728** | 0.00 | 0.00 | 0.13 | 0.13 | FGSG_10313 FGSG_06385 FGSG_16491 FGSG_09903 FGSG_09897 |
| **FGSG_10327** | 0.00 | 0.00 | 0.00 | 0.13 | FGSG_01964 FGSG_06071 |
| **FGSG_05547** | 0.00 | 0.00 | 0.12 | 0.12 | FGSG_10313 FGSG_00719 FGSG_16491 FGSG_09903 FGSG_09897 |
| **FGSG_09857** | 0.25 | 0.00 | 0.00 | 0.12 | FGSG_06385 FGSG_06427 |
| **FGSG_01623** | 0.12 | 0.00 | 0.00 | 0.12 | FGSG_16521 FGSG_06291 |
| **FGSG_10116** | 0.00 | 0.00 | 0.33 | 0.11 | FGSG_01964 FGSG_15914 |
| **FGSG_15838** | 0.00 | 0.00 | 0.22 | 0.11 | FGSG_01964 FGSG_06071 FGSG_15914 |
| **FGSG_05418** | 0.00 | 0.00 | 0.11 | 0.11 | FGSG_06385 FGSG_09897 |
| **FGSG_09408** | 0.00 | 0.00 | 0.11 | 0.11 | FGSG_10313 FGSG_06385 FGSG_16491 FGSG_09903 FGSG_09897 |
| **FGSG_07251** | 0.33 | 0.00 | 0.05 | 0.10 | FGSG_09197 FGSG_10313 FGSG_06385 FGSG_08572 FGSG_13746 FGSG_09897 FGSG_09908 |
| **FGSG_09954** | 0.00 | 0.10 | 0.00 | 0.10 | FGSG_09928 FGSG_01964 |
| **FGSG_16718** | 0.00 | 0.00 | 0.10 | 0.10 | FGSG_10313 FGSG_06385 FGSG_16491 FGSG_09903 FGSG_09897 |
| **FGSG_10387** | 0.00 | 0.11 | 0.00 | 0.10 | FGSG_09928 FGSG_01964 FGSG_06071 FGSG_15914 |
| **FGSG_06206** | 0.00 | 0.00 | 0.08 | 0.10 | FGSG_01555 FGSG_10313 FGSG_06385 FGSG_09897 |
| **FGSG_05455** | 0.00 | 0.00 | 0.10 | 0.09 | FGSG_01665 FGSG_04104 |
| **FGSG_10095** | 0.00 | 0.00 | 0.09 | 0.09 | FGSG_10313 FGSG_06385 FGSG_09897 |
| **FGSG_00362** | 0.00 | 0.00 | 0.09 | 0.09 | FGSG_10313 FGSG_06385 FGSG_16491 FGSG_09903 |
| **FGSG_05734** | 0.00 | 0.00 | 0.09 | 0.09 | FGSG_10313 FGSG_06385 FGSG_16491 FGSG_09903 FGSG_09897 |
| **FGSG_04311** | 0.00 | 0.00 | 0.09 | 0.09 | FGSG_07067 FGSG_01176 |
| **FGSG_16540** | 0.00 | 0.00 | 0.09 | 0.09 | FGSG_10313 FGSG_06385 FGSG_16491 FGSG_09903 FGSG_09897 |
| **FGSG_17594** | 0.00 | 0.00 | 0.09 | 0.09 | FGSG_10313 FGSG_06385 FGSG_16491 FGSG_09903 FGSG_09897 |
| **FGSG_16269** | 0.00 | 0.00 | 0.09 | 0.09 | FGSG_07067 FGSG_01176 |
| **FGSG_08920** | 0.13 | 0.00 | 0.00 | 0.08 | FGSG_06427 FGSG_09897 |
| **FGSG_01842** | 0.00 | 0.00 | 0.08 | 0.08 | FGSG_16491 FGSG_09897 |
| **FGSG_09778** | 0.10 | 0.00 | 0.00 | 0.08 | FGSG_04104 FGSG_09903 FGSG_10114 |
| **FGSG_04947** | 0.06 | 0.00 | 0.13 | 0.08 | FGSG_10313 FGSG_06385 FGSG_16491 FGSG_09903 FGSG_09897 |
| **FGSG_10725** | 0.00 | 0.00 | 0.08 | 0.08 | FGSG_10313 FGSG_06385 FGSG_16491 FGSG_09897 |
| **FGSG_00472** | 0.00 | 0.00 | 0.08 | 0.08 | FGSG_10313 FGSG_06385 FGSG_16491 FGSG_09903 FGSG_09897 |
| **FGSG_16039** | 0.00 | 0.00 | 0.08 | 0.08 | FGSG_01176 FGSG_07067 |
| **FGSG_16006** | 0.00 | 0.00 | 0.08 | 0.08 | FGSG_10313 FGSG_06385 FGSG_16491 FGSG_09897 |
| **FGSG_08635** | 0.00 | 0.00 | 0.08 | 0.07 | FGSG_10313 FGSG_06385 FGSG_16491 FGSG_09897 |
| **FGSG_00469** | 0.00 | 0.00 | 0.07 | 0.07 | FGSG_10313 FGSG_06385 FGSG_09903 FGSG_09897 |
| **FGSG_02083** | 0.00 | 0.12 | 0.00 | 0.07 | FGSG_01964 FGSG_06071 |
| **FGSG_01354** | 0.08 | 0.00 | 0.00 | 0.07 | FGSG_16521 FGSG_06291 |
| **FGSG_01188** | 0.00 | 0.00 | 0.08 | 0.07 | FGSG_10313 FGSG_06385 FGSG_16491 FGSG_09897 |
| **FGSG_10066** | 0.06 | 0.10 | 0.00 | 0.07 | FGSG_09928 FGSG_10313 FGSG_06385 FGSG_01964 FGSG_10384 FGSG_09903 FGSG_15914 |
| **FGSG_04400** | 0.00 | 0.13 | 0.00 | 0.07 | FGSG_06385 FGSG_08769 FGSG_10114 |
| **FGSG_15841** | 0.07 | 0.00 | 0.00 | 0.07 | FGSG_10313 FGSG_06385 FGSG_09903 |
| **FGSG_16947** | 0.06 | 0.00 | 0.06 | 0.07 | FGSG_10313 FGSG_06385 FGSG_16491 FGSG_09903 FGSG_09897 |
| **FGSG_02699** | 0.00 | 0.08 | 0.00 | 0.07 | FGSG_01964 FGSG_15914 |
| **FGSG_01312** | 0.00 | 0.00 | 0.05 | 0.07 | FGSG_10313 FGSG_09614 FGSG_16491 FGSG_09897 |
| **FGSG_00536** | 0.07 | 0.00 | 0.00 | 0.07 | FGSG_10313 FGSG_06385 FGSG_09903 |
| **FGSG_07884** | 0.00 | 0.00 | 0.07 | 0.07 | FGSG_01176 FGSG_07067 |
| **FGSG_17351** | 0.00 | 0.00 | 0.07 | 0.07 | FGSG_01176 FGSG_07067 |
| **FGSG_06266** | 0.07 | 0.00 | 0.00 | 0.07 | FGSG_10313 FGSG_06385 FGSG_09903 |
| **FGSG_04286** | 0.07 | 0.00 | 0.00 | 0.07 | FGSG_10313 FGSG_06385 FGSG_09903 |
| **FGSG_00760** | 0.07 | 0.00 | 0.00 | 0.07 | FGSG_10313 FGSG_06385 FGSG_09903 |
| **FGSG_06878** | 0.06 | 0.08 | 0.06 | 0.07 | FGSG_00385 FGSG_08737 FGSG_10313 FGSG_06385 FGSG_01964 FGSG_09903 FGSG_16491 FGSG_09897 |
| **FGSG_16633** | 0.00 | 0.00 | 0.07 | 0.07 | FGSG_10313 FGSG_06385 FGSG_16491 FGSG_09897 |
| **FGSG_01058** | 0.00 | 0.00 | 0.08 | 0.07 | FGSG_06385 FGSG_16491 FGSG_09897 |
| **FGSG_06940** | 0.00 | 0.00 | 0.06 | 0.06 | FGSG_06385 FGSG_16491 FGSG_09897 |
| **FGSG_08607** | 0.06 | 0.00 | 0.00 | 0.06 | FGSG_10313 FGSG_06385 FGSG_09903 |
| **FGSG_10272** | 0.00 | 0.08 | 0.00 | 0.06 | FGSG_01555 FGSG_01964 FGSG_06071 FGSG_15914 |
| **FGSG_16980** | 0.05 | 0.00 | 0.00 | 0.06 | FGSG_10313 FGSG_06385 FGSG_09903 FGSG_15914 |
| **FGSG_11561** | 0.00 | 0.00 | 0.06 | 0.06 | FGSG_01176 FGSG_07067 |
| **FGSG_00786** | 0.06 | 0.00 | 0.06 | 0.06 | FGSG_10313 FGSG_06385 FGSG_16491 FGSG_09903 FGSG_09897 |
| **FGSG_06324** | 0.00 | 0.00 | 0.06 | 0.06 | FGSG_01176 FGSG_07067 |
| **FGSG_16323** | 0.06 | 0.00 | 0.08 | 0.06 | FGSG_10313 FGSG_06385 FGSG_16491 FGSG_09903 FGSG_09897 |
| **FGSG_11812** | 0.00 | 0.00 | 0.06 | 0.06 | FGSG_06385 FGSG_16491 FGSG_09897 |
| **FGSG_17398** | 0.06 | 0.00 | 0.00 | 0.06 | FGSG_10313 FGSG_06385 FGSG_09903 |
| **FGSG_11614** | 0.06 | 0.00 | 0.00 | 0.06 | FGSG_10313 FGSG_06385 FGSG_09903 |
| **FGSG_13944** | 0.06 | 0.00 | 0.00 | 0.06 | FGSG_10313 FGSG_06385 FGSG_09903 |
| **FGSG_07423** | 0.06 | 0.00 | 0.10 | 0.06 | FGSG_10313 FGSG_06385 FGSG_16491 FGSG_09903 FGSG_09897 |
| **FGSG_16497** | 0.06 | 0.00 | 0.07 | 0.06 | FGSG_10313 FGSG_06385 FGSG_16491 FGSG_09903 FGSG_09897 |
| **FGSG_04910** | 0.06 | 0.00 | 0.00 | 0.06 | FGSG_10313 FGSG_06385 FGSG_09903 |
| **FGSG_06832** | 0.06 | 0.00 | 0.00 | 0.06 | FGSG_10313 FGSG_06385 FGSG_09903 |
| **FGSG_00337** | 0.06 | 0.00 | 0.08 | 0.06 | FGSG_10313 FGSG_06385 FGSG_16491 FGSG_09903 FGSG_09897 |
| **FGSG_08359** | 0.00 | 0.06 | 0.00 | 0.06 | FGSG_00385 FGSG_08737 |
| **FGSG_07329** | 0.06 | 0.00 | 0.11 | 0.06 | FGSG_10313 FGSG_06385 FGSG_16491 FGSG_09903 FGSG_09897 |
| **FGSG_16818** | 0.00 | 0.00 | 0.06 | 0.06 | FGSG_16491 FGSG_09903 FGSG_09897 |
| **FGSG_03146** | 0.06 | 0.00 | 0.00 | 0.06 | FGSG_10313 FGSG_06385 FGSG_09903 |
| **FGSG_09612** | 0.06 | 0.00 | 0.08 | 0.06 | FGSG_10313 FGSG_06385 FGSG_09903 FGSG_16491 FGSG_09897 FGSG_04111 |
| **FGSG_08729** | 0.06 | 0.00 | 0.08 | 0.06 | FGSG_10313 FGSG_06385 FGSG_16491 FGSG_09903 FGSG_09897 |
| **FGSG_17001** | 0.00 | 0.00 | 0.06 | 0.06 | FGSG_16491 FGSG_09903 FGSG_09897 |
| **FGSG_01559** | 0.06 | 0.00 | 0.00 | 0.06 | FGSG_10313 FGSG_06385 FGSG_16491 FGSG_09903 |
| **FGSG_00433** | 0.00 | 0.00 | 0.06 | 0.06 | FGSG_16491 FGSG_09897 |
| **FGSG_05393** | 0.05 | 0.00 | 0.09 | 0.06 | FGSG_10313 FGSG_06385 FGSG_16491 FGSG_09903 FGSG_09897 |
| **FGSG_11878** | 0.06 | 0.00 | 0.00 | 0.06 | FGSG_10313 FGSG_06385 FGSG_09903 |
| **FGSG_02488** | 0.06 | 0.00 | 0.00 | 0.06 | FGSG_10313 FGSG_06385 FGSG_09903 |
| **FGSG_08468** | 0.05 | 0.00 | 0.08 | 0.06 | FGSG_10313 FGSG_06385 FGSG_05388 FGSG_09903 FGSG_16491 FGSG_09897 |
| **FGSG_17186** | 0.00 | 0.00 | 0.07 | 0.06 | FGSG_10313 FGSG_06385 FGSG_09903 FGSG_09897 |
| **FGSG_16412** | 0.06 | 0.00 | 0.00 | 0.06 | FGSG_10313 FGSG_06385 FGSG_09903 |
| **FGSG_08421** | 0.08 | 0.00 | 0.00 | 0.06 | FGSG_09197 FGSG_09895 |
| **FGSG_05737** | 0.06 | 0.00 | 0.00 | 0.06 | FGSG_10313 FGSG_06385 FGSG_09903 |
| **FGSG_15983** | 0.06 | 0.00 | 0.05 | 0.05 | FGSG_10313 FGSG_06385 FGSG_16491 FGSG_09903 FGSG_09897 |
| **FGSG_04054** | 0.05 | 0.00 | 0.10 | 0.05 | FGSG_10313 FGSG_06385 FGSG_16491 FGSG_09903 FGSG_09897 |
| **FGSG_01506** | 0.05 | 0.00 | 0.07 | 0.05 | FGSG_10313 FGSG_06385 FGSG_09903 FGSG_16491 FGSG_09897 FGSG_06874 |
| **FGSG_08135** | 0.00 | 0.00 | 0.05 | 0.05 | FGSG_01176 FGSG_07067 |
| **FGSG_07121** | 0.00 | 0.00 | 0.07 | 0.05 | FGSG_10313 FGSG_06385 FGSG_16491 FGSG_13746 FGSG_09897 |
| **FGSG_16743** | 0.00 | 0.00 | 0.06 | 0.05 | FGSG_10313 FGSG_06385 FGSG_09897 |
| **FGSG_05845** | 0.00 | 0.00 | 0.07 | 0.05 | FGSG_10313 FGSG_06385 FGSG_09903 FGSG_09897 |
| **FGSG_16299** | 0.06 | 0.00 | 0.00 | 0.05 | FGSG_10313 FGSG_06385 FGSG_09903 |
| **FGSG_09739** | 0.00 | 0.11 | 0.00 | 0.05 | FGSG_00385 FGSG_08737 |
| **FGSG_02531** | 0.00 | 0.00 | 0.00 | 0.05 | FGSG_01293 FGSG_01555 |
| **FGSG_09271** | 0.06 | 0.00 | 0.00 | 0.05 | FGSG_09614 FGSG_04104 |
| **FGSG_01271** | 0.00 | 0.00 | 0.07 | 0.05 | FGSG_10313 FGSG_06385 FGSG_16491 FGSG_09897 |
| **FGSG_17687** | 0.00 | 0.00 | 0.06 | 0.05 | FGSG_16491 FGSG_09903 FGSG_09897 |
| **FGSG_04382** | 0.00 | 0.11 | 0.00 | 0.05 | FGSG_01555 FGSG_01964 FGSG_06071 FGSG_09897 |
| **FGSG_03170** | 0.00 | 0.00 | 0.29 | 0.05 | FGSG_01964 FGSG_15914 |
| **FGSG_06970** | 0.00 | 0.00 | 0.06 | 0.05 | FGSG_09903 FGSG_09897 |
| **FGSG_05406** | 0.06 | 0.00 | 0.06 | 0.05 | FGSG_10313 FGSG_06385 FGSG_09903 FGSG_09897 |
| **FGSG_04484** | 0.05 | 0.00 | 0.07 | 0.05 | FGSG_10313 FGSG_06385 FGSG_09903 FGSG_09897 |
| **FGSG_16383** | 0.06 | 0.00 | 0.00 | 0.05 | FGSG_10313 FGSG_06385 FGSG_09903 |
| **FGSG_05778** | 0.00 | 0.00 | 0.00 | 0.05 | FGSG_04104 FGSG_05593 |
| **FGSG_00644** | 0.00 | 0.00 | 0.00 | 0.05 | FGSG_00376 FGSG_10313 |
| **FGSG_10251** | 0.06 | 0.00 | 0.00 | 0.05 | FGSG_09614 FGSG_04104 |
| **FGSG_16988** | 0.00 | 0.00 | 0.05 | 0.05 | FGSG_10313 FGSG_06385 FGSG_09897 |
| **FGSG_05038** | 0.06 | 0.00 | 0.00 | 0.05 | FGSG_09614 FGSG_04104 |
| **FGSG_06959** | 0.00 | 0.00 | 0.05 | 0.05 | FGSG_16491 FGSG_09903 FGSG_09897 |
| **FGSG_07946** | 0.00 | 0.05 | 0.00 | 0.05 | FGSG_01964 FGSG_15914 |
| **FGSG_04618** | 0.07 | 0.00 | 0.00 | 0.05 | FGSG_09614 FGSG_04104 |
| **FGSG_05407** | 0.00 | 0.04 | 0.00 | 0.04 | FGSG_09928 FGSG_01964 FGSG_15914 |
| **FGSG_06209** | 0.00 | 0.00 | 0.00 | 0.04 | FGSG_09928 FGSG_10114 |
| **FGSG_00414** | 0.00 | 0.00 | 0.00 | 0.04 | FGSG_04104 FGSG_00332 |
| **FGSG_01338** | 0.07 | 0.00 | 0.00 | 0.04 | FGSG_10313 FGSG_06385 FGSG_09903 |
| **FGSG_07855** | 0.04 | 0.00 | 0.00 | 0.04 | FGSG_10313 FGSG_06385 FGSG_09903 |
| **FGSG_16828** | 0.05 | 0.00 | 0.09 | 0.04 | FGSG_10313 FGSG_06385 FGSG_09903 |
| **FGSG_08537** | 0.00 | 0.04 | 0.00 | 0.04 | FGSG_00385 FGSG_09928 FGSG_08737 FGSG_15914 |
| **FGSG_05977** | 0.04 | 0.00 | 0.00 | 0.04 | FGSG_09197 FGSG_00719 FGSG_00950 |
| **FGSG_02014** | 0.05 | 0.00 | 0.00 | 0.04 | FGSG_06385 FGSG_09895 |
| **FGSG_17226** | 0.00 | 0.00 | 0.05 | 0.04 | FGSG_16491 FGSG_09903 |
| **FGSG_00559** | 0.07 | 0.03 | 0.00 | 0.04 | FGSG_09197 FGSG_06948 FGSG_09908 FGSG_09895 |
| **FGSG_09690** | 0.05 | 0.00 | 0.00 | 0.04 | FGSG_10313 FGSG_06385 FGSG_09903 |
| **FGSG_16809** | 0.06 | 0.00 | 0.09 | 0.04 | FGSG_10313 FGSG_06385 FGSG_09903 FGSG_16491 |
| **FGSG_06846** | 0.00 | 0.04 | 0.00 | 0.04 | FGSG_00385 FGSG_08737 |
| **FGSG_12297** | 0.00 | 0.00 | 0.40 | 0.04 | FGSG_01964 FGSG_15914 |
| **FGSG_10873** | 0.00 | 0.00 | 0.00 | 0.04 | FGSG_06385 FGSG_10114 |
| **FGSG_05698** | 0.07 | 0.00 | 0.00 | 0.04 | FGSG_09614 FGSG_04104 |
| **FGSG_00408** | 0.00 | 0.00 | 0.04 | 0.04 | FGSG_16491 FGSG_09897 |
| **FGSG_04107** | 0.00 | 0.04 | 0.00 | 0.04 | FGSG_00376 FGSG_06385 |
| **FGSG_00677** | 0.06 | 0.00 | 0.00 | 0.04 | FGSG_10313 FGSG_06385 FGSG_06948 FGSG_09903 |
| **FGSG_02022** | 0.00 | 0.03 | 0.00 | 0.04 | FGSG_08737 FGSG_10313 FGSG_15914 |
| **FGSG_08857** | 0.00 | 0.00 | 0.00 | 0.04 | FGSG_06385 FGSG_10114 |
| **FGSG_16912** | 0.00 | 0.03 | 0.00 | 0.03 | FGSG_00385 FGSG_08737 |
| **FGSG_16028** | 0.06 | 0.00 | 0.00 | 0.03 | FGSG_09614 FGSG_04104 |
| **FGSG_00838** | 0.03 | 0.00 | 0.00 | 0.03 | FGSG_10179 FGSG_09614 FGSG_13746 FGSG_02506 |
| **FGSG_07075** | 0.00 | 0.04 | 0.00 | 0.03 | FGSG_00385 FGSG_08737 |
| **FGSG_06886** | 0.00 | 0.00 | 0.00 | 0.03 | FGSG_06948 FGSG_09895 |
| **FGSG_00071** | 0.00 | 0.38 | 0.00 | 0.03 | FGSG_10397 FGSG_17598 FGSG_03543 |
| **FGSG_02661** | 0.00 | 0.03 | 0.00 | 0.03 | FGSG_08737 FGSG_09908 |
| **FGSG_07335** | 0.03 | 0.03 | 0.00 | 0.03 | FGSG_08737 FGSG_10313 FGSG_06385 FGSG_10825 FGSG_02527 |
| **FGSG_09689** | 0.00 | 0.03 | 0.00 | 0.03 | FGSG_09908 FGSG_09895 |
| **FGSG_10782** | 0.00 | 0.04 | 0.00 | 0.03 | FGSG_00385 FGSG_08737 |
| **FGSG_10002** | 0.00 | 0.03 | 0.00 | 0.03 | FGSG_00376 FGSG_08737 FGSG_06385 |
| **FGSG_07004** | 0.00 | 0.03 | 0.00 | 0.03 | FGSG_08737 FGSG_09908 |
| **FGSG_08543** | 0.00 | 0.04 | 0.00 | 0.03 | FGSG_00385 FGSG_08737 |
| **FGSG_06021** | 0.05 | 0.00 | 0.00 | 0.02 | FGSG_16620 FGSG_17598 |
| **FGSG_05535** | 0.15 | 0.00 | 0.00 | 0.02 | FGSG_09614 FGSG_04104 |
| **FGSG_11626** | 0.00 | 0.02 | 0.00 | 0.02 | FGSG_00385 FGSG_08737 FGSG_10825 |
| **FGSG_11064** | 0.07 | 0.00 | 0.00 | 0.02 | FGSG_10313 FGSG_06385 FGSG_09903 |
| **FGSG_09660** | 0.00 | 0.00 | 0.08 | 0.02 | FGSG_10313 FGSG_06385 FGSG_09903 FGSG_09897 |
| **FGSG_07306** | 0.00 | 0.00 | 0.00 | 0.02 | FGSG_06291 FGSG_06071 |
| **FGSG_10067** | 0.00 | 0.02 | 0.00 | 0.02 | FGSG_09928 FGSG_01964 FGSG_15914 |
| **FGSG_11627** | 0.00 | 0.02 | 0.00 | 0.02 | FGSG_00385 FGSG_08737 |
| **FGSG_09663** | 0.11 | 0.00 | 0.00 | 0.02 | FGSG_13120 FGSG_06874 |
| **FGSG_09586** | 0.00 | 0.02 | 0.00 | 0.02 | FGSG_00385 FGSG_08737 |
| **FGSG_03535** | 0.00 | 0.00 | 0.00 | 0.02 | FGSG_17598 FGSG_03543 |
| **FGSG_03540** | 0.00 | 0.50 | 0.00 | 0.02 | FGSG_17598 FGSG_03543 |
| **FGSG_06895** | 0.00 | 0.00 | 0.00 | 0.02 | FGSG_08737 FGSG_09908 |
| **FGSG_00637** | 0.04 | 0.00 | 0.00 | 0.02 | FGSG_00385 FGSG_08737 FGSG_02506 |
| **FGSG_06847** | 0.00 | 0.02 | 0.00 | 0.02 | FGSG_00376 FGSG_08737 FGSG_06385 |
| **FGSG_08811** | 0.04 | 0.00 | 0.00 | 0.02 | FGSG_09197 FGSG_02506 FGSG_09897 |
| **FGSG_02795** | 0.05 | 0.00 | 0.00 | 0.02 | FGSG_10313 FGSG_06385 FGSG_09903 |
| **FGSG_01888** | 0.00 | 0.02 | 0.00 | 0.02 | FGSG_00385 FGSG_08737 |
| **FGSG_05619** | 0.00 | 0.00 | 0.00 | 0.02 | FGSG_08737 FGSG_04111 |
| **FGSG_09862** | 0.00 | 0.02 | 0.00 | 0.02 | FGSG_09928 FGSG_08737 |
| **FGSG_06921** | 0.00 | 0.02 | 0.00 | 0.01 | FGSG_00385 FGSG_08737 |
| **FGSG_09870** | 0.05 | 0.00 | 0.00 | 0.01 | FGSG_09614 FGSG_04104 |
| **FGSG_06922** | 0.00 | 0.02 | 0.00 | 0.01 | FGSG_00385 FGSG_08737 |
| **FGSG_08560** | 0.00 | 0.00 | 0.00 | 0.01 | FGSG_00376 FGSG_10313 |
| **FGSG_08888** | 0.00 | 0.01 | 0.00 | 0.01 | FGSG_00376 FGSG_08737 |
| **FGSG_01014** | 0.00 | 0.00 | 0.00 | 0.01 | FGSG_08737 FGSG_06385 |
