# Supplementary material for: Network-Based Data Integration for Selecting Candidate Virulence Associated Proteins in the Cereal Infecting Fungus Fusarium graminearum
Source: PLoS One. 2013 Jul 4;8(7):e67926. doi: 10.1371/journal.pone.0067926 (PMC3701590; doi:10.1371/journal.pone.0067926)
Supplement: Table S4 — Estimating the predictive power of the four different networks. (DOCX) [file pone.0067926.s010.docx]

**Table S4: Estimating the predictive power of the four different networks**

| **Feature** | **Integrated** | **PPI** | **Coexpression** | **Sequence-similarity** |
| --- | --- | --- | --- | --- |
| Sensitivity | 1.66% | 1.12% | 0.94% | 1.42% |
| Random control | 0.08% | 0.12% | 0.08% | 0.08% |
| Improvement | 1979% | 967% | 1142% | 1775% |
| Specificity | 94.59% | 81.28% | 100.00% | 100.00% |

The recall / sensitivity was calculated using a jack-knife approach (resampling by removing 10% of seeds and trying to recover them using the remaining ones (see for example ([Vihinen, 2012](#_ENREF_1))). 1000 replicas were done for each network. Random control value was calculated as a chance that a protein picked at random would be a member of a pre-defined set of the same size as the one used to calculate actual sensitivity. Due to the small number of seeds currently available, the coverage in the network is at present too low to accurately estimate how often virulence-associated proteins are connected to each other. However, the recall / sensitivity is still a marked improvement compare to the random model, indicating that there is indeed a non-random pattern of association present in the network.

Vihinen, M. (2012). How to evaluate performance of prediction methods? Measures and their interpretation in variation effect analysis. BMC genomics *13 Suppl 4*, S2.
