# Supplementary material for: Network-Based Data Integration for Selecting Candidate Virulence Associated Proteins in the Cereal Infecting Fungus Fusarium graminearum
Source: PLoS One. 2013 Jul 4;8(7):e67926. doi: 10.1371/journal.pone.0067926 (PMC3701590; doi:10.1371/journal.pone.0067926)
Supplement: Table S5 — Heatmap showing the taxonomic diversity of the matches to the 215 predictions. (DOCX) [file pone.0067926.s011.docx]

**Table S5: Heatmap showing the taxonomic diversity of the matches to the 215 predictions.**

The shading in SEQ, PPI, COEX column shows if the prediction was also made when only links of that type are used. Hits are counted once, at the lowest possible taxonomy level, so for example *F. graminearum* counts are not included in any of the subsequent levels. The *Nectriacaea* column contains all hits from the species in that taxonomy group, excluding those from *F. graminearum.*

| **Gene** | **SEQ** | **PPI** | **COEX** | | **G. zeae** | **Nectriaceae** | **Hypocreales** | **Hypocreomycetidae** | **Sordariomycetes** | **Pezizomycotina** | **Ascomycota** | **Dikarya** | **Fungi** | **Eukaryota** | **Other** |
| --- | --- | --- | --- | --- | --- | --- | --- | --- | --- | --- | --- | --- | --- | --- | --- |
| **FGSG_06878** |  |  | |  |  |  |  |  |  |  |  |  |  |  |  |
| **FGSG_16809** |  |  | |  |  |  |  |  |  |  |  |  |  |  |  |
| **FGSG_00337** |  |  | |  |  |  |  |  |  |  |  |  |  |  |  |
| **FGSG_16497** |  |  | |  |  |  |  |  |  |  |  |  |  |  |  |
| **FGSG_09612** |  |  | |  |  |  |  |  |  |  |  |  |  |  |  |
| **FGSG_15983** |  |  | |  |  |  |  |  |  |  |  |  |  |  |  |
| **FGSG_00786** |  |  | |  |  |  |  |  |  |  |  |  |  |  |  |
| **FGSG_16828** |  |  | |  |  |  |  |  |  |  |  |  |  |  |  |
| **FGSG_07251** |  |  | |  |  |  |  |  |  |  |  |  |  |  |  |
| **FGSG_05393** |  |  | |  |  |  |  |  |  |  |  |  |  |  |  |
| **FGSG_04054** |  |  | |  |  |  |  |  |  |  |  |  |  |  |  |
| **FGSG_08729** |  |  | |  |  |  |  |  |  |  |  |  |  |  |  |
| **FGSG_05406** |  |  | |  |  |  |  |  |  |  |  |  |  |  |  |
| **FGSG_07423** |  |  | |  |  |  |  |  |  |  |  |  |  |  |  |
| **FGSG_04484** |  |  | |  |  |  |  |  |  |  |  |  |  |  |  |
| **FGSG_08468** |  |  | |  |  |  |  |  |  |  |  |  |  |  |  |
| **FGSG_16323** |  |  | |  |  |  |  |  |  |  |  |  |  |  |  |
| **FGSG_01506** |  |  | |  |  |  |  |  |  |  |  |  |  |  |  |
| **FGSG_07329** |  |  | |  |  |  |  |  |  |  |  |  |  |  |  |
| **FGSG_04947** |  |  | |  |  |  |  |  |  |  |  |  |  |  |  |
| **FGSG_16947** |  |  | |  |  |  |  |  |  |  |  |  |  |  |  |
| **FGSG_16693** |  |  | |  |  |  |  |  |  |  |  |  |  |  |  |
| **FGSG_16816** |  |  | |  |  |  |  |  |  |  |  |  |  |  |  |
| **FGSG_06311** |  |  | |  |  |  |  |  |  |  |  |  |  |  |  |
| **FGSG_06684** |  |  | |  |  |  |  |  |  |  |  |  |  |  |  |
| **FGSG_16768** |  |  | |  |  |  |  |  |  |  |  |  |  |  |  |
| **FGSG_16967** |  |  | |  |  |  |  |  |  |  |  |  |  |  |  |
| **FGSG_09715** |  |  | |  |  |  |  |  |  |  |  |  |  |  |  |
| **FGSG_09410** |  |  | |  |  |  |  |  |  |  |  |  |  |  |  |
| **FGSG_07469** |  |  | |  |  |  |  |  |  |  |  |  |  |  |  |
| **FGSG_01410** |  |  | |  |  |  |  |  |  |  |  |  |  |  |  |
| **FGSG_04293** |  |  | |  |  |  |  |  |  |  |  |  |  |  |  |
| **FGSG_07310** |  |  | |  |  |  |  |  |  |  |  |  |  |  |  |
| **FGSG_13314** |  |  | |  |  |  |  |  |  |  |  |  |  |  |  |
| **FGSG_05547** |  |  | |  |  |  |  |  |  |  |  |  |  |  |  |
| **FGSG_17687** |  |  | |  |  |  |  |  |  |  |  |  |  |  |  |
| **FGSG_17728** |  |  | |  |  |  |  |  |  |  |  |  |  |  |  |
| **FGSG_16818** |  |  | |  |  |  |  |  |  |  |  |  |  |  |  |
| **FGSG_17001** |  |  | |  |  |  |  |  |  |  |  |  |  |  |  |
| **FGSG_16633** |  |  | |  |  |  |  |  |  |  |  |  |  |  |  |
| **FGSG_17594** |  |  | |  |  |  |  |  |  |  |  |  |  |  |  |
| **FGSG_00362** |  |  | |  |  |  |  |  |  |  |  |  |  |  |  |
| **FGSG_05734** |  |  | |  |  |  |  |  |  |  |  |  |  |  |  |
| **FGSG_06940** |  |  | |  |  |  |  |  |  |  |  |  |  |  |  |
| **FGSG_01271** |  |  | |  |  |  |  |  |  |  |  |  |  |  |  |
| **FGSG_09408** |  |  | |  |  |  |  |  |  |  |  |  |  |  |  |
| **FGSG_17186** |  |  | |  |  |  |  |  |  |  |  |  |  |  |  |
| **FGSG_16718** |  |  | |  |  |  |  |  |  |  |  |  |  |  |  |
| **FGSG_06970** |  |  | |  |  |  |  |  |  |  |  |  |  |  |  |
| **FGSG_06959** |  |  | |  |  |  |  |  |  |  |  |  |  |  |  |
| **FGSG_16743** |  |  | |  |  |  |  |  |  |  |  |  |  |  |  |
| **FGSG_16988** |  |  | |  |  |  |  |  |  |  |  |  |  |  |  |
| **FGSG_16540** |  |  | |  |  |  |  |  |  |  |  |  |  |  |  |
| **FGSG_07121** |  |  | |  |  |  |  |  |  |  |  |  |  |  |  |
| **FGSG_11812** |  |  | |  |  |  |  |  |  |  |  |  |  |  |  |
| **FGSG_05845** |  |  | |  |  |  |  |  |  |  |  |  |  |  |  |
| **FGSG_16006** |  |  | |  |  |  |  |  |  |  |  |  |  |  |  |
| **FGSG_00472** |  |  | |  |  |  |  |  |  |  |  |  |  |  |  |
| **FGSG_17226** |  |  | |  |  |  |  |  |  |  |  |  |  |  |  |
| **FGSG_10095** |  |  | |  |  |  |  |  |  |  |  |  |  |  |  |
| **FGSG_10725** |  |  | |  |  |  |  |  |  |  |  |  |  |  |  |
| **FGSG_00408** |  |  | |  |  |  |  |  |  |  |  |  |  |  |  |
| **FGSG_00469** |  |  | |  |  |  |  |  |  |  |  |  |  |  |  |
| **FGSG_04053** |  |  | |  |  |  |  |  |  |  |  |  |  |  |  |
| **FGSG_01188** |  |  | |  |  |  |  |  |  |  |  |  |  |  |  |
| **FGSG_09660** |  |  | |  |  |  |  |  |  |  |  |  |  |  |  |
| **FGSG_01312** |  |  | |  |  |  |  |  |  |  |  |  |  |  |  |
| **FGSG_00433** |  |  | |  |  |  |  |  |  |  |  |  |  |  |  |
| **FGSG_07520** |  |  | |  |  |  |  |  |  |  |  |  |  |  |  |
| **FGSG_08635** |  |  | |  |  |  |  |  |  |  |  |  |  |  |  |
| **FGSG_08691** |  |  | |  |  |  |  |  |  |  |  |  |  |  |  |
| **FGSG_06206** |  |  | |  |  |  |  |  |  |  |  |  |  |  |  |
| **FGSG_04418** |  |  | |  |  |  |  |  |  |  |  |  |  |  |  |
| **FGSG_01842** |  |  | |  |  |  |  |  |  |  |  |  |  |  |  |
| **FGSG_01058** |  |  | |  |  |  |  |  |  |  |  |  |  |  |  |
| **FGSG_05418** |  |  | |  |  |  |  |  |  |  |  |  |  |  |  |
| **FGSG_05455** |  |  | |  |  |  |  |  |  |  |  |  |  |  |  |
| **FGSG_15867** |  |  | |  |  |  |  |  |  |  |  |  |  |  |  |
| **FGSG_10470** |  |  | |  |  |  |  |  |  |  |  |  |  |  |  |
| **FGSG_00792** |  |  | |  |  |  |  |  |  |  |  |  |  |  |  |
| **FGSG_00342** |  |  | |  |  |  |  |  |  |  |  |  |  |  |  |
| **FGSG_16005** |  |  | |  |  |  |  |  |  |  |  |  |  |  |  |
| **FGSG_10116** |  |  | |  |  |  |  |  |  |  |  |  |  |  |  |
| **FGSG_16273** |  |  | |  |  |  |  |  |  |  |  |  |  |  |  |
| **FGSG_07504** |  |  | |  |  |  |  |  |  |  |  |  |  |  |  |
| **FGSG_15838** |  |  | |  |  |  |  |  |  |  |  |  |  |  |  |
| **FGSG_02743** |  |  | |  |  |  |  |  |  |  |  |  |  |  |  |
| **FGSG_17038** |  |  | |  |  |  |  |  |  |  |  |  |  |  |  |
| **FGSG_17351** |  |  | |  |  |  |  |  |  |  |  |  |  |  |  |
| **FGSG_11561** |  |  | |  |  |  |  |  |  |  |  |  |  |  |  |
| **FGSG_03170** |  |  | |  |  |  |  |  |  |  |  |  |  |  |  |
| **FGSG_12297** |  |  | |  |  |  |  |  |  |  |  |  |  |  |  |
| **FGSG_06550** |  |  | |  |  |  |  |  |  |  |  |  |  |  |  |
| **FGSG_01949** |  |  | |  |  |  |  |  |  |  |  |  |  |  |  |
| **FGSG_06324** |  |  | |  |  |  |  |  |  |  |  |  |  |  |  |
| **FGSG_04311** |  |  | |  |  |  |  |  |  |  |  |  |  |  |  |
| **FGSG_16039** |  |  | |  |  |  |  |  |  |  |  |  |  |  |  |
| **FGSG_07884** |  |  | |  |  |  |  |  |  |  |  |  |  |  |  |
| **FGSG_08135** |  |  | |  |  |  |  |  |  |  |  |  |  |  |  |
| **FGSG_16269** |  |  | |  |  |  |  |  |  |  |  |  |  |  |  |
| **FGSG_07335** |  |  | |  |  |  |  |  |  |  |  |  |  |  |  |
| **FGSG_00559** |  |  | |  |  |  |  |  |  |  |  |  |  |  |  |
| **FGSG_10066** |  |  | |  |  |  |  |  |  |  |  |  |  |  |  |
| **FGSG_08811** |  |  | |  |  |  |  |  |  |  |  |  |  |  |  |
| **FGSG_02014** |  |  | |  |  |  |  |  |  |  |  |  |  |  |  |
| **FGSG_00637** |  |  | |  |  |  |  |  |  |  |  |  |  |  |  |
| **FGSG_00838** |  |  | |  |  |  |  |  |  |  |  |  |  |  |  |
| **FGSG_16980** |  |  | |  |  |  |  |  |  |  |  |  |  |  |  |
| **FGSG_02010** |  |  | |  |  |  |  |  |  |  |  |  |  |  |  |
| **FGSG_17028** |  |  | |  |  |  |  |  |  |  |  |  |  |  |  |
| **FGSG_01338** |  |  | |  |  |  |  |  |  |  |  |  |  |  |  |
| **FGSG_09271** |  |  | |  |  |  |  |  |  |  |  |  |  |  |  |
| **FGSG_06021** |  |  | |  |  |  |  |  |  |  |  |  |  |  |  |
| **FGSG_01392** |  |  | |  |  |  |  |  |  |  |  |  |  |  |  |
| **FGSG_16028** |  |  | |  |  |  |  |  |  |  |  |  |  |  |  |
| **FGSG_09857** |  |  | |  |  |  |  |  |  |  |  |  |  |  |  |
| **FGSG_01559** |  |  | |  |  |  |  |  |  |  |  |  |  |  |  |
| **FGSG_09778** |  |  | |  |  |  |  |  |  |  |  |  |  |  |  |
| **FGSG_05737** |  |  | |  |  |  |  |  |  |  |  |  |  |  |  |
| **FGSG_06832** |  |  | |  |  |  |  |  |  |  |  |  |  |  |  |
| **FGSG_16299** |  |  | |  |  |  |  |  |  |  |  |  |  |  |  |
| **FGSG_09690** |  |  | |  |  |  |  |  |  |  |  |  |  |  |  |
| **FGSG_16383** |  |  | |  |  |  |  |  |  |  |  |  |  |  |  |
| **FGSG_05698** |  |  | |  |  |  |  |  |  |  |  |  |  |  |  |
| **FGSG_11064** |  |  | |  |  |  |  |  |  |  |  |  |  |  |  |
| **FGSG_09870** |  |  | |  |  |  |  |  |  |  |  |  |  |  |  |
| **FGSG_00677** |  |  | |  |  |  |  |  |  |  |  |  |  |  |  |
| **FGSG_05535** |  |  | |  |  |  |  |  |  |  |  |  |  |  |  |
| **FGSG_09988** |  |  | |  |  |  |  |  |  |  |  |  |  |  |  |
| **FGSG_05038** |  |  | |  |  |  |  |  |  |  |  |  |  |  |  |
| **FGSG_07855** |  |  | |  |  |  |  |  |  |  |  |  |  |  |  |
| **FGSG_04286** |  |  | |  |  |  |  |  |  |  |  |  |  |  |  |
| **FGSG_10251** |  |  | |  |  |  |  |  |  |  |  |  |  |  |  |
| **FGSG_09535** |  |  | |  |  |  |  |  |  |  |  |  |  |  |  |
| **FGSG_08607** |  |  | |  |  |  |  |  |  |  |  |  |  |  |  |
| **FGSG_02795** |  |  | |  |  |  |  |  |  |  |  |  |  |  |  |
| **FGSG_08920** |  |  | |  |  |  |  |  |  |  |  |  |  |  |  |
| **FGSG_08421** |  |  | |  |  |  |  |  |  |  |  |  |  |  |  |
| **FGSG_04618** |  |  | |  |  |  |  |  |  |  |  |  |  |  |  |
| **FGSG_11878** |  |  | |  |  |  |  |  |  |  |  |  |  |  |  |
| **FGSG_05977** |  |  | |  |  |  |  |  |  |  |  |  |  |  |  |
| **FGSG_15841** |  |  | |  |  |  |  |  |  |  |  |  |  |  |  |
| **FGSG_04910** |  |  | |  |  |  |  |  |  |  |  |  |  |  |  |
| **FGSG_01354** |  |  | |  |  |  |  |  |  |  |  |  |  |  |  |
| **FGSG_05512** |  |  | |  |  |  |  |  |  |  |  |  |  |  |  |
| **FGSG_03146** |  |  | |  |  |  |  |  |  |  |  |  |  |  |  |
| **FGSG_02488** |  |  | |  |  |  |  |  |  |  |  |  |  |  |  |
| **FGSG_06266** |  |  | |  |  |  |  |  |  |  |  |  |  |  |  |
| **FGSG_01623** |  |  | |  |  |  |  |  |  |  |  |  |  |  |  |
| **FGSG_00536** |  |  | |  |  |  |  |  |  |  |  |  |  |  |  |
| **FGSG_09663** |  |  | |  |  |  |  |  |  |  |  |  |  |  |  |
| **FGSG_00460** |  |  | |  |  |  |  |  |  |  |  |  |  |  |  |
| **FGSG_17398** |  |  | |  |  |  |  |  |  |  |  |  |  |  |  |
| **FGSG_16412** |  |  | |  |  |  |  |  |  |  |  |  |  |  |  |
| **FGSG_00760** |  |  | |  |  |  |  |  |  |  |  |  |  |  |  |
| **FGSG_11614** |  |  | |  |  |  |  |  |  |  |  |  |  |  |  |
| **FGSG_13944** |  |  | |  |  |  |  |  |  |  |  |  |  |  |  |
| **FGSG_10822** |  |  | |  |  |  |  |  |  |  |  |  |  |  |  |
| **FGSG_15757** |  |  | |  |  |  |  |  |  |  |  |  |  |  |  |
| **FGSG_07004** |  |  | |  |  |  |  |  |  |  |  |  |  |  |  |
| **FGSG_07075** |  |  | |  |  |  |  |  |  |  |  |  |  |  |  |
| **FGSG_11626** |  |  | |  |  |  |  |  |  |  |  |  |  |  |  |
| **FGSG_08543** |  |  | |  |  |  |  |  |  |  |  |  |  |  |  |
| **FGSG_07946** |  |  | |  |  |  |  |  |  |  |  |  |  |  |  |
| **FGSG_11627** |  |  | |  |  |  |  |  |  |  |  |  |  |  |  |
| **FGSG_06847** |  |  | |  |  |  |  |  |  |  |  |  |  |  |  |
| **FGSG_10272** |  |  | |  |  |  |  |  |  |  |  |  |  |  |  |
| **FGSG_06922** |  |  | |  |  |  |  |  |  |  |  |  |  |  |  |
| **FGSG_04107** |  |  | |  |  |  |  |  |  |  |  |  |  |  |  |
| **FGSG_04382** |  |  | |  |  |  |  |  |  |  |  |  |  |  |  |
| **FGSG_09689** |  |  | |  |  |  |  |  |  |  |  |  |  |  |  |
| **FGSG_04400** |  |  | |  |  |  |  |  |  |  |  |  |  |  |  |
| **FGSG_10067** |  |  | |  |  |  |  |  |  |  |  |  |  |  |  |
| **FGSG_09739** |  |  | |  |  |  |  |  |  |  |  |  |  |  |  |
| **FGSG_06921** |  |  | |  |  |  |  |  |  |  |  |  |  |  |  |
| **FGSG_10782** |  |  | |  |  |  |  |  |  |  |  |  |  |  |  |
| **FGSG_09862** |  |  | |  |  |  |  |  |  |  |  |  |  |  |  |
| **FGSG_00883** |  |  | |  |  |  |  |  |  |  |  |  |  |  |  |
| **FGSG_02022** |  |  | |  |  |  |  |  |  |  |  |  |  |  |  |
| **FGSG_03540** |  |  | |  |  |  |  |  |  |  |  |  |  |  |  |
| **FGSG_10002** |  |  | |  |  |  |  |  |  |  |  |  |  |  |  |
| **FGSG_08537** |  |  | |  |  |  |  |  |  |  |  |  |  |  |  |
| **FGSG_02699** |  |  | |  |  |  |  |  |  |  |  |  |  |  |  |
| **FGSG_01228** |  |  | |  |  |  |  |  |  |  |  |  |  |  |  |
| **FGSG_09586** |  |  | |  |  |  |  |  |  |  |  |  |  |  |  |
| **FGSG_10387** |  |  | |  |  |  |  |  |  |  |  |  |  |  |  |
| **FGSG_08888** |  |  | |  |  |  |  |  |  |  |  |  |  |  |  |
| **FGSG_09954** |  |  | |  |  |  |  |  |  |  |  |  |  |  |  |
| **FGSG_00071** |  |  | |  |  |  |  |  |  |  |  |  |  |  |  |
| **FGSG_10167** |  |  | |  |  |  |  |  |  |  |  |  |  |  |  |
| **FGSG_02661** |  |  | |  |  |  |  |  |  |  |  |  |  |  |  |
| **FGSG_05407** |  |  | |  |  |  |  |  |  |  |  |  |  |  |  |
| **FGSG_01548** |  |  | |  |  |  |  |  |  |  |  |  |  |  |  |
| **FGSG_08359** |  |  | |  |  |  |  |  |  |  |  |  |  |  |  |
| **FGSG_02083** |  |  | |  |  |  |  |  |  |  |  |  |  |  |  |
| **FGSG_06846** |  |  | |  |  |  |  |  |  |  |  |  |  |  |  |
| **FGSG_01888** |  |  | |  |  |  |  |  |  |  |  |  |  |  |  |
| **FGSG_03534** |  |  | |  |  |  |  |  |  |  |  |  |  |  |  |
| **FGSG_16912** |  |  | |  |  |  |  |  |  |  |  |  |  |  |  |
| **FGSG_10808** |  |  | |  |  |  |  |  |  |  |  |  |  |  |  |
| **FGSG_06886** |  |  | |  |  |  |  |  |  |  |  |  |  |  |  |
| **FGSG_05619** |  |  | |  |  |  |  |  |  |  |  |  |  |  |  |
| **FGSG_08560** |  |  | |  |  |  |  |  |  |  |  |  |  |  |  |
| **FGSG_10873** |  |  | |  |  |  |  |  |  |  |  |  |  |  |  |
| **FGSG_06209** |  |  | |  |  |  |  |  |  |  |  |  |  |  |  |
| **FGSG_08857** |  |  | |  |  |  |  |  |  |  |  |  |  |  |  |
| **FGSG_07306** |  |  | |  |  |  |  |  |  |  |  |  |  |  |  |
| **FGSG_00414** |  |  | |  |  |  |  |  |  |  |  |  |  |  |  |
| **FGSG_05778** |  |  | |  |  |  |  |  |  |  |  |  |  |  |  |
| **FGSG_01014** |  |  | |  |  |  |  |  |  |  |  |  |  |  |  |
| **FGSG_06895** |  |  | |  |  |  |  |  |  |  |  |  |  |  |  |
| **FGSG_00644** |  |  | |  |  |  |  |  |  |  |  |  |  |  |  |
| **FGSG_10327** |  |  | |  |  |  |  |  |  |  |  |  |  |  |  |
| **FGSG_02531** |  |  | |  |  |  |  |  |  |  |  |  |  |  |  |
| **FGSG_03535** |  |  | |  |  |  |  |  |  |  |  |  |  |  |  |
