# Supplementary material for: Network-Based Data Integration for Selecting Candidate Virulence Associated Proteins in the Cereal Infecting Fungus Fusarium graminearum
Source: PLoS One. 2013 Jul 4;8(7):e67926. doi: 10.1371/journal.pone.0067926 (PMC3701590; doi:10.1371/journal.pone.0067926)
Supplement: Table S6 — Prediction of FGSG_09715, FGSG_07251 and FGSG_10066 as virulence associated proteins. (DOCX) [file pone.0067926.s012.docx]

**Table S6**: The 3 prediction of FGSG_09715, FGSG_07251 and FGSG_10066 as predicted virulence proteins on the basis of links to 7 seeds. Annotation is from FGDB at MIPS.

| **Predicted virulence protein (and MIPS annotation)** | **Seeds on which the prediction is based with phenotype [], and MIPS annotation; Phenotype symbols are rv=reduced virulence, lp=loss of pathogenicity** | | |
| --- | --- | --- | --- |
|  | **Linked by predicted PPI** | **Linked by co-expression** | **Linked by sequence similarity** |
| **FGSG_09715** probable carbon repressor protein 1 |  | **FGSG_06071** [lp] conserved hypothetical protein | **FGSG_07928** [rv] related to transcription factor RGM1  **FGSG_08617** [rv] related to zinc finger protein  **FGSG_01022** [rv] related to metalloregulatory protein  **FGSG_01350** [rv] related to zinc finger protein crol gamma  **FGSG_06871** [rv] related to cutinase G-box binding protein  **FGSG_01341** [rv] related to calcineurin responsive zinc-finger protein |
| **FGSG_07251** PKAC probable cAMP-dependent protein kinase | **FGSG_13746** [rv] related to NOT3 - general negative regulator of transcription, subunit 3  **FGSG_09908** [rv] probable cAMP-dependent protein kinase regulatory chain (mcb)  **FGSG_08572** [lp] related to GDP/GTP exchange factor Rom2p  **FGSG_09197** [rv] probable 3-hydroxy-3-methylglutaryl-coenzyme A reductase |  | **FGSG_09897** [rv] probable serine/threonine protein kinase (SNF1)  **FGSG_06385** [lp] FMK1 pathogenicity MAP kinase 1  **FGSG_10313** [rv] MGV1 MAP kinase   \|  \| \| --- \| |
| **FGSG_10066** probable casein kinase I cki2 | **FGSG_10384** [rv] probable MBP1 - transcription factor, subunit of the MBF factor  **FGSG_10313** [rv] MGV1 MAP kinase  **FGSG_09903** [lp] FST7 probable MAP kinase kinase  **FGSG_06385** [lp] FMK1 pathogenicity MAP kinase 1 | **FGSG_01964** [rv] probable chitin synthase  **FGSG_15914** [rv] probable chitin synthase  **FGSG_09928** [rv] GzSYN2 related to putative snare protein syn |  |
