# Supplementary material for: Network-Based Data Integration for Selecting Candidate Virulence Associated Proteins in the Cereal Infecting Fungus Fusarium graminearum
Source: PLoS One. 2013 Jul 4;8(7):e67926. doi: 10.1371/journal.pone.0067926 (PMC3701590; doi:10.1371/journal.pone.0067926)
Supplement: Table S7 — Mapping the data from Wang et al to the integrated network. (DOCX) [file pone.0067926.s013.docx]

**Table S7: Mapping the data from Wang et al (2011) to the integrated network.**

1. Seed proteins in Wang et al dataset

reduced: FGSG_05484 FGSG_10313 FGSG_06385 FGSG_09903 FGSG_09897

1. Predicted proteins in Wang et al dataset

essential**:** FGSG_05393 FGSG_07855 FGSG_07423 FGSG_09660 FGSG_06959 FGSG_09408 FGSG_04054 FGSG_08731 FGSG_07409 FGSG_00677 FGSG_01271 FGSG_10725 FGSG_05845

reduced**:** FGSG_07251 FGSG_05418 FGSG_04053 FGSG_02795 FGSG_04947 FGSG_00472 FGSG_06878 FGSG_03284 FGSG_08468 FGSG_09612 FGSG_07329 FGSG_08635 FGSG_01312 FGSG_10095 FGSG_08906 FGSG_05547 FGSG_07295 FGSG_08691 FGSG_00362 FGSG_05734 FGSG_04484 FGSG_10066 FGSG_11812 FGSG_04770 FGSG_01188

unaffected**:** FGSG_11614 FGSG_02399 FGSG_10591 FGSG_08729 FGSG_06832 FGSG_04418 FGSG_02838 FGSG_06970 FGSG_13944 FGSG_05549 FGSG_03146 FGSG_06206 FGSG_12149 FGSG_07121 FGSG_01559 FGSG_01058 FGSG_05406 FGSG_05135 FGSG_02488 FGSG_01506 FGSG_06940 FGSG_00786 FGSG_00337 FGSG_00469 FGSG_03132

Wang C, Zhang S, Hou R, Zhao Z, Zheng Q, et al. (2011) Functional analysis of the kinome of the wheat scab fungus Fusarium graminearum. PLoS pathogens 7: e1002460.
