# Supplementary material for: Network-Based Data Integration for Selecting Candidate Virulence Associated Proteins in the Cereal Infecting Fungus Fusarium graminearum
Source: PLoS One. 2013 Jul 4;8(7):e67926. doi: 10.1371/journal.pone.0067926 (PMC3701590; doi:10.1371/journal.pone.0067926)
Supplement: Table S8 — The publically available F. graminearum microarray gene expression datasets used in this study. (DOCX) [file pone.0067926.s014.docx]

**Table S8**: The publically available *F. graminearum* microarray gene expression datasets used in this study.

| **Experiment** | **PLEXdb accession**^♦^ | **Reference** |
| --- | --- | --- |
| ***In planta*** |  |  |
| Infected barley and wheat spikes | FG1, FG15 | ([Guldener et al., 2006](#_ENREF_3); [Lysoe et al., 2011b](#_ENREF_7)) |
| Wheat stem post anthesis | FG12, FG16 | ([Stephens et al., 2008](#_ENREF_10); [Guenther et al., 2009](#_ENREF_2)) |
| ***In vitro*** |  |  |
| Perithecial development | FG5 | ([Hallen et al., 2007](#_ENREF_4)) |
| Asexual spore germination | FG7 | ([Seong et al., 2008](#_ENREF_8)) |
| Media experiments, trichodiene treatment, DON induction | FG2, FG10, FG14 | ([Guldener et al., 2006](#_ENREF_3); [Gardiner et al., 2009](#_ENREF_1); [Seong et al., 2009](#_ENREF_9)) |
| Mutant characterization | FG6, FG11, FG13, | ([Hallen and Trail, 2008](#_ENREF_5); [Seong et al., 2009](#_ENREF_9); [Lysoe et al., 2011a](#_ENREF_6)) |

^♦^ [www.PLEXdb.org](http://www.PLEXdb.org) ([Wise et al., 2007](#_ENREF_11))

Gardiner, D.M., Kazan, K., and Manners, J.M. (2009). Novel genes of *Fusarium graminearum* that negatively regulate deoxynivalenol production and virulence. Mol Plant-Microbe Interact *22*, 1588-1600.

Guenther, J.C., Hallen-Adams, H.E., Bucking, H., Shachar-Hill, Y., and Trail, F. (2009). Triacylglyceride metabolism by *Fusarium graminearum* during colonization and sexual development on wheat. Mol Plant-Microbe Interact *22*, 1492-1503.

Guldener, U., Seong, K.Y., Boddu, J., Cho, S., Trail, F., Xu, J.R., Adam, G., Mewes, H.W., Muehlbauer, G.J., and Kistler, H.C. (2006). Development of a *Fusarium graminearum* Affymetrix GeneChip for profiling fungal gene expression *in vitro* and in *planta*. Fungal Genet Biol *43*, 316-325.

Hallen, H.E., Huebner, M., Shiu, S.H., Guldener, U., and Trail, F. (2007). Gene expression shifts during perithecium development in *Gibberella zeae* (anamorph *Fusarium graminearum*), with particular emphasis on ion transport proteins. Fungal Genet Biol *44*, 1146-1156.

Hallen, H.E., and Trail, F. (2008). The L-type calcium ion channel *cch1* affects ascospore discharge and mycelial growth in the filamentous fungus *Gibberella zeae* (anamorph *Fusarium graminearum*). Eukaryot Cell *7*, 415-424.

Lysoe, E., Pasquali, M., Breakspear, A., and Kistler, H.C. (2011a). The transcription factor FgStuAp influences spore development, pathogenicity, and secondary metabolism in *Fusarium graminearum*. Mol Plant-Microbe Interact *24*, 54-67.

Lysoe, E., Seong, K.Y., and Kistler, H.C. (2011b). The transcriptome of *Fusarium graminearum* during the infection of wheat. Mol Plant-Microbe Interact *24*, 995-1000.

Seong, K., Zhao, X., Xu, J., Guldener, U., and Kistler, H. (2008). Conidial germination in the filamentous fungus Fusarium graminearum. Fungal Genetics and Biology *45*, 389 - 399.

Seong, K.Y., Pasquali, M., Zhou, X., Song, J., Hilburn, K., McCormick, S., Dong, Y., Xu, J.R., and Kistler, H.C. (2009). Global gene regulation by *Fusarium* transcription factors Tri6 and Tri10 reveals adaptations for toxin biosynthesis. Mol Microbiol *72*, 354-367.

Stephens, A.E., Gardiner, D.M., White, R.G., Munn, A.L., and Manners, J.M. (2008). Phases of infection and gene expression of *Fusarium graminearum* during crown rot disease of wheat. Molecular plant-microbe interactions : MPMI *21*, 1571-1581.

Wise, R., Caldo, R., Hong, L., Shen, L., Cannon, E., and Dickerson, J. (2007). BarleyBase/PLEXdb: A unified expression profiling database for plants and plant pathogens. In Methods Mol Biol, D. Edwards, ed. (Totowa, NJ, Humana Press), pp. 347 - 363.
